# Grain yield and adaptation of spring wheat to Norwegian growing conditions is driven by allele frequency changes at key adaptive loci discovered by genome-wide association mapping

**DOI:** 10.1007/s00122-023-04424-9

**Published:** 2023-08-17

**Authors:** Tomasz Mroz, Jon Arne Dieseth, Morten Lillemo

**Affiliations:** 1 Department of Plant Sciences, Norwegian University of Life Sciences, 1432 Ås, Norway; 2 Graminor, AS, Bjørke Gård, Hommelstadvegen 60, 2322 Ridabu, Norway

## Abstract

**Key message:**

Adaptation to the Norwegian environment is associated with polymorphisms in the *Vrn-A1* locus. Historical selection for grain yield in Nordic wheat is associated with *TaGS5-3A* and *TaCol-5* loci.

**Abstract:**

Grain yields in Norwegian spring wheat increased by 18 kg ha^−1^ per year between 1972 and 2019 due to introduction of new varieties. These gains were associated with increments in the number of grains per spike and extended length of the vegetative period. However, little is known about the genetic background of this progress. To fill this gap, we conducted genome-wide association study on a panel consisting of both adapted (historical and current varieties and lines in the Nordics) and important not adapted accessions used as parents in the Norwegian wheat breeding program. The study concerned grain yield, plant height, and heading and maturity dates, and detected 12 associated loci, later validated using independent sets of recent breeding lines. Adaptation to the Norwegian cropping conditions was found to be associated with the *Vrn-A1* locus, and a previously undescribed locus on chromosome 1B associated with heading date. Two loci associated with grain yield, corresponding to the *TaGS5-3A* and *TaCol-5* loci, indicated historical selection pressure for high grain yield. A locus on chromosome 2A explained the tallness of the oldest accessions. We investigated the origins of the beneficial alleles associated with the wheat breeding progress in the Norwegian material, tracing them back to crosses with Swedish, German, or CIMMYT lines. This study contributes to the understanding of wheat adaptation to the Norwegian growing conditions, sheds light on the genetic basis of historical wheat improvement and aids future breeding efforts by discovering loci associated with important agronomic traits in wheat.

**Supplementary Information:**

The online version contains supplementary material available at 10.1007/s00122-023-04424-9.

## Introduction

Hexaploid bread wheat (*Triticum aestivum* L.) is among the essential and most traded staple foods, providing around 20% of daily protein and calorie intake for approximately 4.5 billion people worldwide (Braun et al. 2010). The annual genetic gain in wheat grain yield (GY) must be increased from the current levels to 1.0–1.6% per year to meet the food demands of the projected global population in 2050. However, due to current and future challenges posed by climate change, such as reduced soil health, change in temperature, and erratic rainfall, the acceleration of the annual genetic gains remains a challenge (FAO 2017).

Today, wheat cropping in Norway is an integral part of the country’s sustainability policy, despite being relatively small in size compared to the other Nordic countries (0.33 Mt in Norway vs. 2.67 Mt in Sweden, 0.84 Mt in Finland and 4.47 Mt in Denmark) (FAOSTAT, data from 2010 to 2020; https://www.fao.org/faostat). From 1972 to 2019, wheat breeding in Norway increased GY by 17.8 kg ha^−1^ (0.34%) per year and prolonged grain filling and vegetation periods by 2 and 3 days, respectively, showing better adaptation of new cultivars to the changing climate (Mróz et al. 2022). However, to enable further genetic gains in Norwegian spring wheat by utilizing genomics-driven breeding approaches, such as marker-assisted selection (MAS) or genomic prediction (GP), a detailed association study of important agronomic and quality traits in the current breeding material is needed.

The development of genotyping technologies has allowed for the gradual replacement of simple sequence repeats (SSR) and diversity array technology (DArT) markers with much more numerous single nucleotide polymorphism (SNP) markers, which provides an effective mean to identify associations between various traits and loci through genome-wide association studies (GWAS) (Jin et al*.* 2016; Li et al*.* 2016; Quan et al*.* 2021). The ability of GWAS to utilize natural germplasm collections allowed for bypassing the time needed to develop biparental populations for more traditional linkage analyses (Shi et al*.* 2017). Various GWAS models have been developed to successfully account for population structure and spurious associations while reducing computational effort, including MLM (Zhang et al. 2010), CMLM, MLMM (Segura et al. 2012), SUPER (Wang et al. 2014), GBLUP (Zhang et al. 2007) and FarmCPU (Liu et al. 2016). However, except for the stable QTL (quantitative trait locus/loci) with significant effects, loci discovered by GWAS for many traits are often population or environment specific. To tackle this challenge, an alternative method of QTL mapping, meta-QTL analysis (MQTL), was proposed, collecting independently discovered QTL from different populations and environments on a consensus map (Miao et al. 2022), pointing out regions consistently associated with a trait.

The success of a wheat crop is largely determined by adaptation, consisting of genes for phenology and development and their interactions with one another and the environment (Hyles et al. 2020). In order to reach maximum kernel size and number, wheat must develop biomass and flower at the time of optimal seasonal conditions (Trethowan 2014). One of the key components of adaptation is the vernalization, defined as a requirement of low temperatures to flower. The vernalization requirement is mainly determined by the *Vrn1* loci on the long arm of chromosome 5 with copies in all the three wheat sub-genomes. Winter wheats usually carry the winter alleles at all three homoeologous loci, while it is not uncommon for spring wheat to carry the winter allele at *Vrn-A1* to extend the vegetative growth period (Yan et al. 2003, 2004, 2006). Another critical aspect of wheat adaptation is photoperiod sensitivity, largely determined by alleles of the *Ppd1* (*Photoperiod1*) gene, with homoeologous copies on chromosomes 2A, 2B, and 2D (Ramirez et al. 2018). When both photoperiod and vernalization requirements are fully satisfied, there are still relatively minor differences in flowering time. Such differences observed in populations with fixed major flowering time genes *Vrn1* or *Ppd1* led to the discovery of *Eps* (*Earliness *per se) genes. *Eps* genes usually have small effects (Griffiths et al. 2009) and are critical for fine-tuning of developmental patterns (Lewis et al. 2008); however, the genetics of *Eps* are still not as well understood as the mechanisms associated with the *Ppd1* or *Vrn1* loci (Ochagavía et al. 2019).

There have been many loci under historical selection pressure in wheat due to human-driven breeding efforts to improve yield, disease resistance, and other desirable traits. The *Rht* genes, the main genes of the Green Revolution, contributed to dramatic gains in GY by controlling plant height, and thus harvest index (Liu et al. 2017). Selection for favorable alleles in the *Rht-B1 and -D1* loci led to the development of semi-dwarf varieties with increased GY, suitable for intensive agriculture (Wang et al. 2014). Another selected locus is *TaGW2*, involved in regulating grain weight in wheat (Zhang et al. 2018). Recently, a locus on chromosome 3A—*TaGS5-3A*—was discovered to be under historical selection pressure in Chinese wheat, associated with increments in grain size (Wang et al*.* 2016).

Significant progress in GY was discovered in Norwegian spring wheat over the last five decades due to introduction of new varieties (Mróz et al. 2022), but little is known about its genetic basis. The Norwegian growing environment is distinct, and the genetic causes for genotype adaptation are largely unknown. To fill these gaps, the aims of this study were: (i) to detect genomic regions associated with grain yield, plant height, days to heading and days to maturity; (ii) to discover genomic regions associated with genotype adaptation under Norwegian growing conditions; and (iii) to explore genetic explanations for the historical breeding progress in grain yield and plant height in Norwegian spring wheat.

## Materials and methods

### Plant material

The NMBU spring wheat panel, consisting of 301 hexaploid spring wheat varieties and breeding lines, was used for the primary association study. The same panel was recently used for genetic analyses of *Fusarium* head blight (Nannuru et al. 2022), Septoria nodorum blotch (Lin et al. 2022) and yellow rust (Lin et al. 2023) resistance. The collection encompasses 186 Norwegian, 40 Swedish, and 37 lines from CIMMYT, with several additional lines from Australia, Brazil, Canada, Czech Republic, Denmark, Finland, France, Germany, Netherlands, Poland, Russia, Slovakia, South Africa, Switzerland, UK, and the USA. Varieties from Norway or Sweden are adapted to the local growing conditions, while the remaining lines form the “exotic” (not adapted) part of the panel. The whole set encompasses historically significant and current varieties, covering the highlights of the last decades in wheat breeding in the Nordics and worldwide, representing a broad genetic and phenotypic diversity. This collection also contains historical varieties on the Norwegian market, described in Mróz et al. (2022). This collection is referred hereafter to as the main panel.

An independent set of 889 current breeding lines was used for QTL validation. This collection originated from the commercial spring wheat breeding program of Graminor AS (Ridabu, Norway) and is hereafter referred to as the validation panels. Not every genotype was tested in each season/location combination due to different genotype content and number in every field season (ranging from 90 to 397 lines, detailed overview in Table S3). The validation panels are considered adapted to the Norwegian growing conditions due to their origin.

### Field trials

Field trials were carried out for the main panel during field seasons 2015–2021 at Vollebekk Research Station (Norway, Ås, 59° 39′ N, 10° 45′ E) and Staur farm (Norway, Stange, 60° 43′ N, 11° 06′ E). Those locations represent Norway’s two main and economically important wheat-growing areas: the somewhat warmer and milder climate of south-eastern Norway and the slightly colder and temperate climate of inland Norway, respectively.

The trials were fertilized at sowing with 120 kg N ha^−1^ of compound NPK fertilizer (YaraMila 22-3-10) and planted each season on the break of April and May in both locations (exact planting dates in Table S1). Following germination, trials were kept disease and weed free according to local management practices using herbicides (Tripali [active ingredients: florasulam + metsulfuron-methyl + tribenuron-methyl] and Duplosan Meko [mekoprop]) and fungicides (Proline [prothioconazole], Aviator Xpro [bixafen + prothioconazole], Forbel [fenpropimorph] and/or Comet Pro [pyraklostrobin]) in doses tailored to the needs. Irrigation was applied in case of drought that could affect the growth of the plants. Alleys within the trials were created by spraying Glyphosate shortly after seedling emergence. The trials were harvested each season toward the end of August after all varieties had reached full ripeness.

Season 2018 in both locations was marked by very little rainfall and high temperatures during the early growth stages of the plants (almost no rain from May to mid-June, Figs. S1, S2, Tables S4, S5), which, despite irrigation efforts, caused severe damage to the trials. This damage reduced GY by nearly 60% and caused many plant agronomical characteristics to be abnormal (data not shown). Therefore, we excluded the 2018 field season at both locations from the analysis.

Field trials of the validation panels were carried out following the same procedures as for the main panel in field seasons 2019–2022 at Staur and Vollebekk locations.

### Field trial design

The trials were designed as an alpha-lattice with two replicates per genotype, block size of 6, and positions of every accession randomized each year in each location. Each column was planted with buffer variety at its start and end to eliminate border effects. Each field trial plot was 5 × 1.5 m in size at harvest, with gaps between the plots of 30 cm and a central alley of 1 m. Not every variety was tested for the main panel in each season/location. The number of genotypes tested varied from 100 to 295 per season/location, with 301 and 295 unique accessions in Vollebekk and Staur, respectively (Table S2).

### Phenotyping data

The collection was phenotyped for days to heading (DH), days to maturity (DM), grain yield (GY), and plant height (PH). Not every trait was phenotyped in every environment (season/location combination) (Table 1).

**Table 1:** Overview of when the phenotyped traits were captured in each location for the main panel

Trait	Abb	Unit	Vollebekk	Staur
Days to maturity	DM	dss	2015, 2016, 2017, 2019, 2020, 2021	2016, 2017, 2019, 2020
Grain yield	GY	g m^−2^	2015, 2016, 2017, 2019, 2020, 2021	2016, 2017, 2019, 2020
Days to heading	DH	dss	2015, 2016, 2017, 2019, 2020, 2021	2017, 2019, 2020
Plant height	PH	cm	2015, 2016, 2017, 2019, 2020, 2021	2019, 2020

dss = days since sowing

DH and DM were assessed by recording when approximately 50% of the tillers in an experimental plot had reached the respective stage. GY was measured by harvesting and threshing the trial plots, drying the yield until approximately 13.5% moisture, weighing it, and recalculating it to g per m^2^. PH was assessed by measuring the distance between the ground and the top of spikes (excluding awns, if present) for a random tiller sample when plants reached their final height.

Data for plots that lodged early in the season was removed due to the heavy impact on their development. If lodging occurred later in the season (close to physiological maturity), data were double-checked for consistency and possible impact on the traits and judged if they should be included in the dataset.

### Statistical analysis of the field trials

For each trait, three types of genotypic means (lsmeans) were calculated: location/season (field trial—environment) means, location mean (all seasons in one location), and a global mean, where all the locations and seasons were combined.

As it was common to observe extra spatial variability within the trials (due to soil gradients) that could not have been captured by blocking, an additional covariate was introduced (columns) into the models to correct it.

The lsmeans were calculated using packages “lme4” and “lmerTEST” and custom scripts in R, version 4.2.1.

Field trial lsmeans were calculated using the mixed model (1):

1 $$P_{ilmn} = \mu + g_{i} + R_{l} + R:B_{lm} + C_{n} + e_{ilmn}$$

Cross-season lsmeans for each location were calculated using the mixed model (2):

2 $$P_{iklmn} = \mu + g_{i} + Y_{k} + Y:R_{kl} + Y:R:B_{klm} + Y:C_{kn} + e_{iklmn}$$

Global means (cross-season, cross-location) were calculated using the mixed model (3):

3 $$P_{ijklmn} = \mu + g_{i} + L_{j} + L:Y_{jk} + L:Y:R_{jkl} + L:Y:R:B_{jklm} + L:Y:C_{jkn} + e_{ijklmn}$$

where ${P}_{ijklmn}$ is the phenotype (trait) value for genotype ${g}_{i}$ in location ${L}_{j}$ in season ${Y}_{k}$, planted within replicate ${R}_{l}$, block ${B}_{m}$ and column ${C}_{n}$. Small letters denote fixed effects, capitalized letters denote random effects and “:” denotes nesting of effects. $\mu$ is the general mean and $e$ denotes the error, $IID(0,{\sigma }_{e}^{2}$).

Field trial (season/location), location, and an overall mean (across all field trials) are hereafter referred to as environment, location, and global mean, respectively.

For the validation panel, only environment means were calculated due to varying genotype content in each environment.

Broad-sense heritability (H^2^) was used to assess data quality (replicability), calculated for individual trials using Eq. (4):

4 $$H^{2} = \frac{{\sigma_{G}^{2} }}{{\sigma_{G}^{2} + \sigma_{e}^{2} }}$$

where ${\sigma }_{g}^{2}$ is the genotypic variance and ${\sigma }_{e}^{2}$ is the error variance. Variance components for Eq. (4) were estimated using package “lme4” using a fully random model (5):

5 $$P_{i} = G_{i} + e_{i}$$

where ${P}_{i}$ is the phenotype (trait) value of genotype ${G}_{i}$ and ${e}_{i}$ is the error term, $IID(0,{\sigma }_{e}^{2})$.

Data visualization was performed in R using packages “ggplot2”, “ggpubr” and “ggsci”.

Pearson’s correlation analysis of environments was performed on sets of either environment, location, or global means. Correlation analysis of phenotypes was conducted on the “global” means of the investigated phenotypes.

Environment comparison was carried out by comparing the mean estimates of a phenotype in a given environment (environmental means).

### Genotypic data

Samples were prepared and genotyped with the TraitGenetics 25 K SNP chip as described in Nannuru et al. (2022).

The physical positions of the markers were determined using the chip’s documentation, and markers not mapped to any physical chromosome position were placed on a fictional chromosome Un.

Markers were filtered, leaving only the ones with less than 10% missing data and minor allele frequency (MAF) larger than 0.05. Heterozygous markers were treated as missing data. After the quality check, the dataset contained 19,874 high-quality markers mapped to sub-genomes A (7999), B (7905), and D (2111) on chromosomes 1A (1156), 1B (1147), 1D (391), 2A (1232), 2B (1377), 2D (437) 3A (1074), 3B (1336), 3D (256), 4A (699), 4B (602), 4D (111), 5A (1340), 5B (1406), 5D (311), 6A (1126), 6B (1082), 6D (319), 7A (1372), 7B (955), 7D (285) and Un (1859).

### Population structure, GWAS, and linkage disequilibrium

The main panel exhibits a strong population structure due to the presence of “adapted” and “exotic” groups of lines; therefore, additional correction for population structure was applied by including principal genomic components in the model. Due to poor adaptation, the exotic lines often exhibit unusual phenotypes under Norwegian growing conditions. To mitigate the risk of confounding SNPs with line adaptation and to discover possible sources of adaptation, two series of GWAS were carried out for each phenotype: on the whole collection, and adapted part only. Detailed description of the population structure of the main panel can be found in Nannuru et al. (2022).

GWAS and LD (linkage disequilibrium) analysis were performed using GAPIT v3.2 (Wang and Zhang 2021) in R version 4.2.1. GWAS was performed on a series of phenotypes for each trait: all environment means, location means, and global mean, computed as described in the section Statistical analysis of the field trials.

### Detecting peak markers using FarmCPU method

Several models’ performance was considered, including CMLM (Zhang et al. 2010), MLMM (Segura et al. 2012), SUPER (Wang et al. 2014), GBLUP (Zhang et al. 2007), and FarmCPU (Liu et al. 2016).

The FarmCPU method was chosen to detect peak markers based on superior accordance with the null hypothesis and stronger signal compared to the other methods and its ability to “distillate” markers in a given significant locus by providing fewer MTAs (marker-trait associations), but with a stronger signal. FarmCPU is a multi-locus model based on the MLM method, which relies on iterative and alternative use of fixed and random effect models to minimize the proportion of false positives. Markers are tested one by one using random models. Then the resulting significant associations are used as covariates in a fixed model (random models allow for avoidance of the overfitting issue present with fixed effect models). FarmCPU is well-suited for highly quantitative trait analysis (Liu et al. 2016).

MTAs for each trait were considered based on Bonferroni—corrected *p* values (effective threshold of − log10(p) = 5.6). MTAs that crossed the threshold at least for the global mean, and two other environments were reported. However, all traits are highly quantitative and controlled by many small effect loci; therefore, the Bonferroni correction of *p* values can be too conservative (Haikka et al. 2020). Therefore, a less stringent criterion of *p* < 0.001 was applied. A region was considered meaningful if two or more SNPs within 5 Mbp distance appeared significant for the global mean and at least two different environments/means.

#### Expanding QTL regions around peak markers

Consistent peak markers discovered using the FarmCPU method (section above) were used to anchor haplotypes. A window of 40 Mbp (chosen based on linkage disequilibrium, Fig. S3) around a peak marker’s position was studied using the MLM method (also implemented in GAPIT v3.2). Markers were considered based on appearing significant (− log_10_(p) > 3) for the global mean and at least two environments/means. Peak markers alongside significant markers in the window were used to construct haplotypes. The search for MTAs with the MLM method was carried out in the adapted and complete datasets, similarly to the FarmCPU method.

Individual markers and QTL haplotypes were tested for their associations with the respective traits on the global mean without population structure correction in both the full main panel and its adapted part. Comparison between marker alleles and among haplotype alleles was performed using Tukey’s Honestly Significant Difference (HSD) posthoc test, α = 0.05. Rare haplotype alleles (appearing in less than ten accessions) were discarded from the analysis due to insufficient statistical power to detect their associations. The proportion of phenotype variance explained by each SNP and each putative QTL was estimated using linear models and reported separately as a percentage in the whole panel and its adapted part.

### Analysis of allele frequency over time

Varieties in the adapted part of the main panel were assigned a breeding line year of creation by analyzing their documentation. For varieties for which it was only possible to establish the year they were released to the market, seven years were subtracted to obtain the year of creation (based on the average time it took from variety creation and release to the market in the collection). It was possible to establish the year of creation (YOC) for 180 lines in the adapted part of the main panel, assigned to the following seven periods: pre-1960 (2 lines), 1960–1969 (4), 1970–1985 (6), 1986–1995 (9), 1996–2005 (24), 2006–2010 (41), and 2011 onwards (94). Allele frequency for each discovered haplotype and MTA in GWAS was calculated for each period and analyzed for trends over the years.

### SNP and QTL effect validation

Effects of the significant SNPs discovered in the main panel (full panel and its adapted part) were tested using an independent set of varieties and breeding lines originating from Graminor AS (Ridabu, Norway) spring wheat breeding program (section Plant Material). The validation panels were genotyped using TraitGenetics 25 K SNP chip, identically as described for the main panel.

Field trial data were analyzed using mixed models as described in the section “Statistical analysis of the field trials” section without calculating cross-environment means due to different genotype composition each year. Associations of markers and haplotypes were tested against each season’s genotypic means without correcting for population structure using Tukey’s Honestly Significant Difference (HSD) posthoc test, α = 0.05. Rare haplotype alleles (appearing in less than ten accessions) were discarded from the analysis due to a lack of statistical power to detect their associations.

## Results

### Phenotype data

Differences between the two field trial locations are visible for every studied trait. Lines in the main panel grown in Staur, on average, tend to head nine days earlier (DH), mature 2.5 days earlier (DM), have higher grain yield by 90 g m^−2^ (GY) and reach lower plant height by 4.5 cm (PH) compared to Vollebekk. The trials conducted in Staur exhibit higher variability in DH, DM, and GY (Table 2).

**Table 2:** Descriptive statistics and variance explained by cultivar adaptation for days to heading (DH), days to maturity (DM), grain yield (GY), and plant height (PH) in all the studied environments (Env) and means

Env/mean	DH (dss)			DM (dss)			GY (gm^−2^)			PH (cm)		
	Avg	SD	Var_AD_	Avg	SD	Var_AD_	Avg	SD	Var_AD_	Avg	SD	Var_AD_
V2015	70.73	1.35	4.29	126.42	2.96	12.55	426	91	53.67	81.70	9.90	9.36
V2016	62.00	1.59	2.68	109.83	3.80	4.71	387	77	53.38	73.09	8.92	1.22
S2016	–	–	–	100.92	4.14	18.13	497	114	30.00	–	–	–
V2017	59.19	1.68	6.39	99.32	1.96	0.27	440	75	53.48	77.98	8.60	3.56
S2017	58.04	2.10	29.61	120.82	5.88	18.85	789	118	54.27	–	–	–
V2019	68.04	1.25	0.67	108.49	1.63	1.09	601	55	28.57	95.99	8.68	0.34
S2019	47.88	2.46	14.94	99.80	6.02	18.04	530	65	17.20	86.86	5.15	0.06
V2020	66.48	1.44	0.17	117.76	3.27	5.62	649	82	40.05	83.79	6.67	0.44
S2020	61.64	1.36	2.15	112.28	1.37	0.37	592	81	56.72	70.24	5.25	0.14
V2021	64.41	1.74	0.01	104.46	2.85	11.40	540	69	46.27	86.12	7.76	0.78
Vmean	65.14	1.39	0.47	111.13	2.32	10.55	509	72	63.68	82.98	7.65	1.70
Smean	55.93	1.66	16.65	108.58	3.69	13.76	600	97	56.25	78.50	5.03	0.14
Gmean	60.75	1.42	3.69	110.12	2.74	13.63	548	81	64.24	81.86	7.05	1.28

Avg—mean value, SD—standard deviation, Var_AD_—fraction of variance explained by cultivar adaptation (in percent). For single field trials (environments): letter designates location (V—Vollebekk, S—Staur) and number denotes season (year). Vmean—cross-season trait means in location Vollebekk, Smean—cross-season trait means in location Staur, Gmean—cross-season, cross-location Global means

**Fig. 1:**
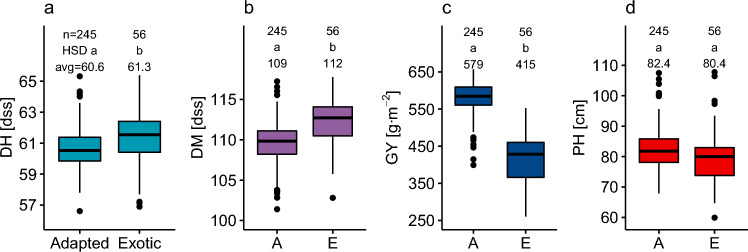
Phenotypic differences among the genotypes due to line adaptation in **a** days to heading, **b** days to maturity, **c** grain yield, and **d** plant height. Groups with the same letter are not significantly different (HSD test, α = 0.05). *A* adapted, *E* exotic lines; *n* number of records in the group, *avg* average phenotype value in the group

The most substantial difference between the exotic and adapted lines of the main panel is seen for GY, with a severe reduction of GY in non-adapted lines accounting for 64% of the total phenotypic variance in GY. Adapted lines also tend to head earlier, mature earlier, and be slightly taller, but with the grouping explaining much less of the phenotypic variance (Table 2, Fig. 1).

Traits analyzed in this study exhibited high heritability (H^2^ > 0.63). DH showed the highest heritability (on average, 0.84), followed by DM, GY, and PH (0.63). PH achieved the highest variability in H^2^ (0.39–0.9) compared to other traits (differences around 0.2) in different environments. Heritability estimates from Vollebekk and Staur experiment sites were comparable (0.73 and 0.69, respectively) except for PH (0.78 and 0.47 from Vollebekk and Staur, respectively) (Table 3).

**Table 3:** Broad-sense heritability estimates for each trait and each field trial

Trait	Vollebekk							Staur					Avg
	2015	2016	2017	2019	2020	2021	Avg	2016	2017	2019	2020	Avg	
DM	0.77	0.62	0.51	0.69	0.66	0.75	0.67	0.73	0.58	0.61	0.42	0.59	0.63
GY	0.85	0.61	0.49	0.68	0.57	0.73	0.66	0.61	0.69	0.51	0.53	0.59	0.63
DH	0.86	0.67	0.84	0.81	0.79	0.89	0.81	–	0.88	0.92	0.77	0.86	0.84
PH	0.85	0.83	0.66	0.90	0.59	0.86	0.78	–	–	0.55	0.39	0.47	0.63

*DM* days to maturity, *GY* grain yield, *DH* days to heading, *PH* plant height

Trial means for all traits are highly positively correlated (r > 0.5) across all-season/location combinations, except for seasons 2015 in Vollebekk (lower correlations for GY and DH) (Fig. S4-7)

**Fig. 2:**
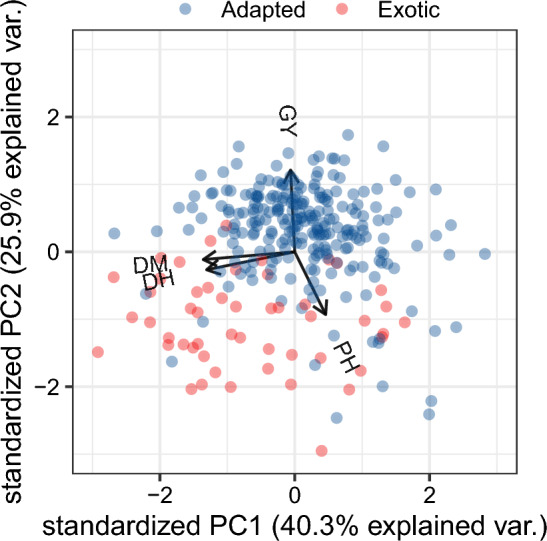
Principal Component Analysis (PCA) of the main panel based on phenotypical data. Color indicates line adaptation: blue—adapted, red—exotic (not adapted). *GY* grain yield, *DM* days to maturity, *DH* days to heading, *PH* plant height

PCA of phenotypic data separates adapted from the exotic part of the panel (Fig. 2).

**Fig. 3:**
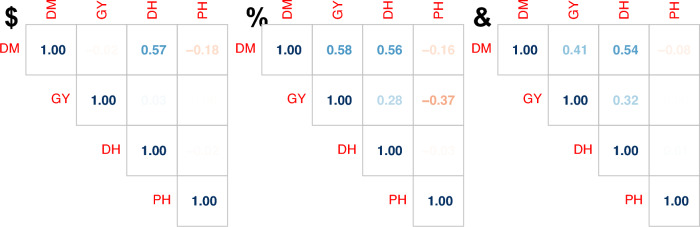
Pearson correlation matrices for genotypic means of **a** all accessions, **b** adapted accessions and **c** exotic accessions. *DM* days to maturity, *GY* grain yield, *DH* days to heading, *PH* plant height

The strong population structure could be seen in the lack of a significant relationship between DM and GY for the whole panel (Fig. 3a) and its presence within both the adapted and exotic parts of the collection (Fig. 3bc). PH showed a weak negative correlation with GY for adapted lines (Fig. 3b), not observed in the entire panel or its exotic part.

**Fig. 4:**
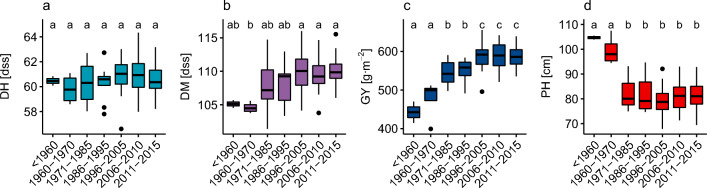
Changes in the traits over the seven periods (x axis): days to heading (**a**, DH, blue), days to maturity (**b**, DM, violet), grain yield (**c**, GY, dark blue), and plant height (**d**, PH, red). Periods with the same letter are not significantly different (HSD test, α = 0.05)

Accessions for which it was possible to establish the year of creation (YOC, 180 lines) were assigned to the following seven periods: pre-1960 (2 lines), 1960–1969 (4), 1970–1985 (6), 1986–1995 (9), 1996–2005 (24), 2006–2010 (41), and 2011 onwards (94). Significant trends over the years using this grouping can be seen for all traits except days to heading. Lines belonging to the time periods from 1996–2005 onwards mature later than lines created in the 1960s, and old accessions (until 1970) are significantly taller than later lines. For GY, there has been consecutive increases with the lines created during 1971–1995 yielding significantly higher than older ones, but less than the lines created after 1995 (Fig. 4).

### GWAS results

GWAS analyses were conducted for each trait for the whole panel and its adapted parts. QQ and Manhattan plots are available in Fig. S8-15 and S16-23, respectively.

A total of 13 consistent and highly significant MTAs were detected using the FarmCPU method, pointing to 12 QTL regions across all sub-genomes (three in A subgenome, five in B, and two in D). Two QTL were discovered for DH on chromosomes 1B and 7B, two for DM on chromosomes 6B and 6D, three for GY on chromosomes 3A, 5A, and 7B, and five for PH on chromosomes 2A, 4A, 4B, 4D, and 6B. All these regions met the stringent Bonferroni threshold for the most significant marker, except for the plant height QTL on 4B (*QHt.nmbu-4B*), where the peak markers were discovered using the criterion of *p* < 0.001 across the global mean and two other environments and two MTAs within a 5 Mb window. The number of significant SNPs (detected using FarmCPU and MLM methods) in each QTL varied from 1 to 18, and no QTL was associated with more than one trait (Table 4, S6).

**Table 4:** QTL regions discovered in the study for days to heading (DH), days to maturity (DM), grain yield (GY), and plant height (PH), their genomic locations (Chr, Span in Mbp), number of significant markers in the locus (n), peak marker(s), QTL effects—full difference between homozygotes—in the full dataset (E_F_) and in the adapted part (E_A_) in units respective to the traits (Table 1), trait variance explained in the full dataset (%PVE_F_) and in the adapted part (%PVE_A_), and summary of validation results (Valid.) corresponding to Table S7

Trait	QTL	Chr	Span	n	Peak marker(s)	^a^E_F_	^a^%PVE_F_	^b^E_A_	^b^%PVE_A_	^c^Valid
DH	*QHd.nmbu-1B*	1B	1–2	2	BS00022180_51	2.04	14.1	Mono	Mono	Rare
	*QHd.nmbu-7B*	7B	606	1	BobWhite_c3541_152	0.91	11.4	0.91	11.4	Rare
DM	*QMat.nmbu-6B*	6B	132–136	10	Kukri_rep_c71420_511	5.93	25.8	4.85	24.8	+
	*QMat.nmbu-6D*	6D	6	1	BS00022523_51	0.82	8.80	0.81	10.0	+
GY	*QYld.nmbu-3A*	3A	267	1	BS00110129_51	38.1	22.0	16.4	14.3	+
	*QYld.nmbu-5A*	5A	683–708	8	BobWhite_c8266_227	192.6	67.2	Ns	Ns	+
	*QYld.nmbu-7B*	7B	701–703	6	BS00083578_51	106.2	16.3	83.6	30.9	+
PH	*QHt.nmbu-2A*	2A	524–543	5	AX-95095516	7.91	9.21	7.97	12.6	Rare
	*QHt.nmbu-4A*	4A	570–603	3	CAP11_c3631_75	9.77	9.36	5.45	9.08	+
	*QHt.nmbu-4B*	4B	13–59	18	TG0010a; TG0010b	8.20	27.7	7.22	23.9	+
	*QHt.nmbu-4D*	4D	19–26	3	BobWhite_s64797_152	8.29	32.5	9.32	42.9	+
	*QHt.nmbu-6B*	6B	202	1	Ra_c10469_616	2.00	5.21	1.25	2.14	+

^a^Value estimated without correcting for population structure using a linear model in the full dataset

^b^Value estimated without correcting for population structure using a linear model in the adapted dataset

^c^Validation of effects without correcting for population structure in sets of independent lines in a total of six environments using a linear model. +—at least one SNP in the region showed significant effect (HSD test, α = 0.05) in one of the validation sets. Rare—all markers had MAF < 0.05 (estimation not reliable), Mono—QTL region monomorphic in the validation panels (estimation not possible)

Two QTL regions were detected for DH on chromosomes 1B and 7B, explaining 14 and 11% of the variance and with two and one-day effects, respectively. Both consisted of a few SNPs (two and one, respectively). *QHd.nmbu-1B* was strongly associated with population structure, as it showed a significant effect only in the whole dataset due to a lack of haplotype diversity in the adapted part. *QHd.nmbu-7B* consistently showed the same effect and proportion of variance explained in both datasets (Table 4, Table S6), indicating a lack of association with line adaptation. *QHd.nmbu-1B* and *QHd.nmbu-7B* had low MAF in the validation sets, making the estimation of their effects unreliable (Table S6).

For DM, two QTL regions were detected on chromosomes 6B and 6D, consistently associated in the complete and adapted datasets. *QMat.nmbu-6B* consisted of 10 markers, while *QMat.nmbu-6D* included only one marker discovered using the FarmCPU method. *QMat.nmbu-6B* showed a considerable effect of around five days, explaining approximately 25% of the variance in both datasets. Similarly, *QMat.nmbu-6D* was detected consistently across the datasets, explaining 9 to 10% of the variance, with a minor effect of 0.8 days. No adaptation-specific QTL was discovered for DM (Table 4, Table S6). Validation of both *QMat.nmbu-6B* and *QMat.nmbu-6D* was successful, with multiple MTAs being confirmed for *QMat.nmbu-6B* and the one MTA comprising *QMat.nmbu-6D* (BS00022523_51, Table S7).

Three QTL regions were detected for GY with effects (without correcting for population structure) ranging from 38 to 193 g m^−2^ and explaining between 16 and 67% of the variance in GY in the complete set. *QYld.nmbu.5A* is associated with line adaptation, showing a very high effect and proportion of variance explained in the entire dataset (193 g m^−2 ^and 67%, respectively) while exhibiting no significant effect in the adapted set. Also, all the MTAs of *QYld.nmbu.5A* were discovered in the entire dataset. However, the remaining two QTL (*QYld.nmbu.3A* and *QYld.nmbu.7B*) were consistently associated with GY in both datasets, with smaller effects (Tables 4, S6). Validation confirmed significant associations of at least one marker in each QTL region associated with GY (1 SNP for *QYld.nmbu.3A*, 3 for *QYld.nmbu.5A*, and 2 for *QYld.nmbu.7B*) (Table S7).

The highest number of QTL regions (5) was detected for PH, on chromosomes 2A, 4A, 4B, 4D, and 6B. All the QTL regions for PH consisted of more than 3 SNPs except for *QHt.nmbu-6B* (only one peak marker, discovered using the FarmCPU method). The QTL had effects ranging from 2 to 10 cm and explained 5 to 33% of the variance in PH in the entire dataset. None of the QTL appeared to be adaptation-specific, as all showed significant and comparable effects in both adapted and complete datasets. As the presence of exotic lines increases the variance in PH considerably (Fig. 1), it is remarkable that *QHt.nmbu-4D*’s proportion of variance explained and effect are higher in the adapted than in the entire dataset (Table 4). Validation confirmed the associations of *QHt.nmbu-4A*, *QHt.nmbu-4B*, *QHt.nmbu-4D*, and *QHt.nmbu-6B* with high confidence (multiple markers in the loci appeared significantly associated in multiple validation sets in multiple environments). *QHt.nmbu-2A* could not be validated due to residual minor allele frequencies of the SNPs comprising it in the validation sets (Table S7).

### Adaptation to Norwegian growing conditions

**Fig. 5:**
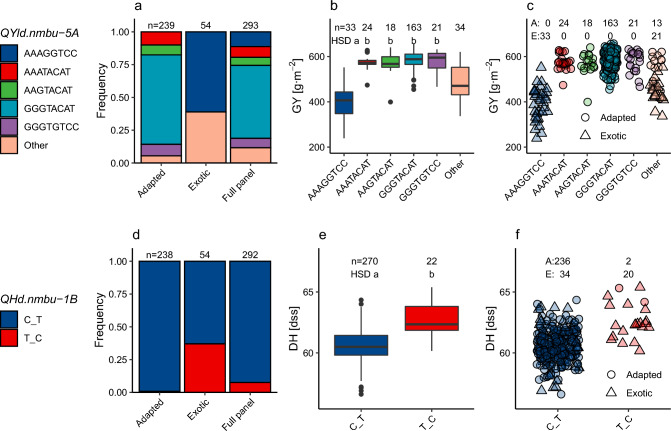
Loci associated with adaptation to the Norwegian growing conditions: *QYld.nmbu-5A* (**a**–**c**) and *QHd.nmbu-1B* (**d**–**f**). Allele frequencies in the full panel and adapted and exotic parts (**a**, **d**), haplotype analysis of the loci (**b**, **e**), and presence of alleles in adapted and exotic parts of the panel (**c**, **f**). Comparison among the alleles was performed using Tukey’s HSD test. Alleles with the same letter are not significantly different (α = 0.05). Only alleles present in more than ten lines were considered for *QHd.nmbu-1B*. For *QYld.nmbu-5A*, alleles with low frequencies (in less than ten lines) were gathered into the “Other” bin, and not included in the HSD test. Association of *QYld.nmbu-5A* haplotypes with DH is shown in Fig. S24

Two QTL regions associated with DH and GY (*QHd.nmbu-1B* and *QYld.nmbu-5A*) consistently appeared highly significant for their respective traits in the entire dataset while showing no polymorphism/effect in the adapted part of the dataset (Table 4, Fig. 5). It was therefore reasonable to consider these QTL as pointers to genomic regions associated with genotype adaptation to the Norwegian growing conditions.

The *QYld.nmbu-5A* (683–708 Mbp) region (Table 4) showed an extremely high effect of 192 g m^2^ (almost 2 t ha) and captured 67% of GY variance in the entire dataset. The high proportion of variance explained by the QTL makes it likely that *QYld.nmbu-5A* is the leading cause for the observed differences between the adapted and exotic lines. Interestingly, even though *QYld.nmbu-5A* showed no significant effect in the main panel’s adapted part, three SNPs belonging to it showed significant association with GY in the validation panels, however, with effects not nearly as high as in the entire main panel (Table S7). *QYld.nmbu-5A* is effectively represented by five haplotypes (occurring in more than ten lines) and several underrepresented variants (present in less than ten lines). The allele associated with lower GY (AAAGGTCC) occurs exclusively in exotic lines, while high-GY alleles are present in adapted lines only. The rare alleles are present in 13 and 21 adapted and exotic lines, respectively. Exotic lines carrying one of the underrepresented alleles also exhibit lower GY (Fig. 5a–c). An analogous haplotype analysis of *QYld.nmbu-5A* revealed its significant association also with DH, which could not be detected by GWAS (Fig. S24).

*QHd.nmbu-1B* (1–2 Mb), capturing 14% of the variance in DH in the entire dataset (Table 4), is a likely candidate to contribute to line adaptation concerning DH. However, the average proportion of DH variance explained by line adaptation is lower (4%, Table 2). Not all exotic lines are identical in this locus, being a consequence of various origins of the exotic lines. The early haplotype (CT) is present in almost all adapted lines, and in more than half of the exotic accessions. The late haplotype (TC) occurs in only two adapted lines, and twenty exotic lines (Fig. 5d–f). *QHd.nmbu-1B* also exhibited almost no polymorphism in the validation sets.

### Breeding progress in Norwegian spring wheat

**Fig. 6:**
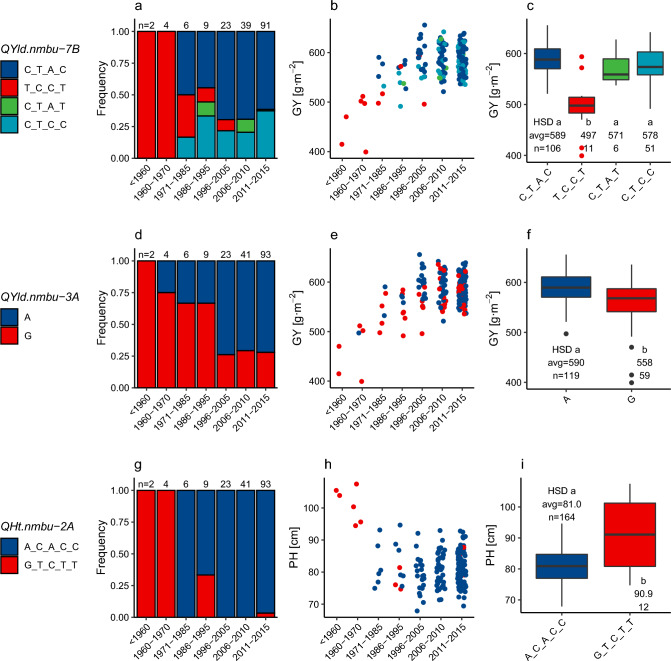
Columns, from left to right: allele frequency over time, relationships between QTL alleles and their respective trait across the periods, and comparison among QTL alleles and the respective trait for *QYld.nmbu-7B* (**a**–**c**), *QYld.nmbu-3A* (**d**–**f**), and *QHt.nmbu-2A* (**g**–**i**). Rare alleles occurring in less than 5 lines were removed from the analysis. Comparisons among QTL alleles were performed using HSD (Honestly Significant Difference) test. Alleles with the same letter are not significantly different (α = 0.05)

Three of the detected QTL showed noticeable change in allele frequency over the periods: *QYld.nmbu-7B*, *QYld.nmbu-3A*, and *QHt.nmbu-2A* (Fig. 6).

*QYld.nmbu-7B* was consistently discovered in both adapted and complete datasets and captured nearly 31% of the variance in GY in the adapted lines (Tables 2, ) This QTL is represented in the adapted lines by four haplotypes: three with similar, positive effects, and one allele with strong negative effect (TCCT) explained mostly by SNP variation in the first marker (BS00083578_51) (Fig. 6c). The negative effect allele is present in all old accessions (before 1970). After 1971, its frequency decays gradually to zero in the 2006–2015 bracket, replaced by either positive effect allele. Two outliers carrying the negative effect allele are visible with high GY values, comparable to lines carrying one of the favorable effect alleles (Fig. 6c). *QYld.nmbu-7B* frequencies also align well with the observed increase in GY over the periods. The diversity in the validation panels in the *QYld.nmbu-7B* locus was generally low. The new breeding lines were dominated by the favorable effect alleles, limiting the prospects of exploiting this locus for further GY improvement. However, SNPs belonging to *QYld.nmbu-7B* still showed a significant association with GY in the validation panels (Table S7).

Another locus showing allele frequency change is *QYld.nmbu-3A* but not as radically as *QYld.nmbu-7B*. *QYld.nmbu-3A* comprises a single SNP, consistently detected using FarmCPU and MLM methods (Table S6) and explained 14 and 22% of GY variance in adapted and complete datasets, respectively (Table 4). This locus is polymorphic in all periods (except the oldest lines) and shows a decay in negative allele (G) frequency over time. However, it still retains a degree of polymorphism in the most recent breeding lines (Fig. 6d–f). Although this region comprised only a single SNP, it was still significantly associated with GY in the validation sets and is a potential GY improvement source in future breeding (Table S7).

Allele frequency changing over the periods can also be observed for *QHt.nmbu-2A*, represented in the adapted lines effectively by only two alleles, despite five significant MTAs comprising this locus. The lines dating before 1970 all carry the tall allele (GTCTT), while the later accessions mostly carry the shortening allele (ACACC). However, the tall allele could still be found in 3 lines created between 1986 and 1995 and two more recent breeding lines (Fig. 6g–i). The locus also showed almost no diversity in the validation panels (Table S7).

By investigating the allele frequencies of SNPs in the validation panels (representing the most recent germplasm in Norway), it can be observed that SNP alleles associated with increased GY and DM are visibly dominating in the population (most lines carry the favorable effect alleles) (Table S7).

## Discussion

Crossing adapted germplasm with exotic parents is an essential source of variation and valuable alleles (Reynolds et al. 2009) and has been actively used in Norwegian spring wheat breeding by introducing mainly CIMMYT lines into breeding programs, yielding many market-important varieties (Lillemo and Dieseth 2011). Therefore, analyzing only lines adapted to the distinct Nordic environments would appear incomplete for this study. The exotic lines pose a statistical challenge, as those often exhibit extreme phenotypes paired with distinct genetic backgrounds. However, the statistical model used—FarmCPU—did effectively account for the population structure, as judged by the QQ plots. Although the population structure can be perceived as a shortcoming of the association panel, it allows us to explore the genetic basis of line adaptation to the Nordic growing environment.

The number of discovered regions associated with the traits is relatively modest due to the stringent significance criterion applied. However, considering the presence of strong population structure and different backgrounds of lines in the studied population, it was necessary to reduce the risk of committing type 1 errors at the cost of a higher number of false negatives. The purpose of this study was to pinpoint the most important loci for each of the traits. Despite their highly quantitative nature, it was possible to discover several large-effect QTL, explaining the most important genetic variability in the traits in the panel. Apart from the two loci associated with adaptation, 10 QTL regions were consistently discovered in the entire panel and its adapted part.

Line adaptation to the Norwegian growing conditions is most visible for GY: more than half of the variance in GY can be attributed to line adaptation, with much smaller proportions for the other investigated traits. By comparing significant loci for the entire panel and its adapted part alone, two loci were highly significant in the whole panel, with no effect in its adapted part, hinting that those regions may either play a role in adaptation to the Nordic growing environment, be a remnant of line origin, or be random (spurious). *QYld.nmbu-5A*’s polymorphism explains a significant part of the variance in GY (similar to the variance explained by line adaptation), is comprised of multiple SNPs, and coincides with the chromosomal location of the *Vernalization1* locus *Vrn-A1*. Vernalization response is a well-described, crucial component of genotype adaptation to particular growing conditions (reviewed by Hyles et al. (2020)). Lines of diverse origins will carry different alleles in this locus, favorable in their environments of origin. Considering the causative link between *Vrn-A1* and GY and line adaptation, it is reasonable to expect differences in flowering time. Indeed, the non-adapted lines, on average, head later than the adapted ones, but the differences are not as strongly pronounced as for GY. Despite that no signal of the *Vrn-A1* locus was detected for DH in GWAS due to the stringent threshold, *QYld.nmbu-5A* still shows a significant effect on DH, as revealed by haplotype analysis. Additionally, in another round of late-planted trials of the same panel (sowing date in late June), the heading and flowering time differences were much more pronounced (data not shown), indicating that the vernalization genes are playing a crucial role in adaptation to the Nordic growing environment. The fact that *QYld.nmbu-5A* has effect on both GY and DH is convincing that GY benefits from phenological adaptation and not from some other linked gene in the vicinity of the *Vrn-A1* locus.

The second locus associated with line adaptation, *QHd.nmbu-1B*, captures over 14% of the variance in DH in the whole set. *QHd.nmbu-1B*’s peak marker (BS00022180_51) has been previously discovered for DH in the same panel (Sørensen 2016), was found significant for drought stress adaptation (Kamruzzaman 2022) and was associated with sensitivity to the *Parastagonospora nodurum* Tox1 effector (Cockram et al. 2015). However, to the best of the authors’ knowledge, does not align with any previously described locus for days to heading, except for the previous study on the same panel referred to above. The fact that line adaptation in this study is mainly associated with GY and, to a smaller extent, DH, highlights the importance of phenology for adaptation to the relatively short Norwegian growing season. Other important traits not considered directly in this study but documented elsewhere include the ability to withstand lodging and pre-harvest sprouting as well as disease resistance (Lillemo and Dieseth 2011).

Unlike most studies on historical genetic gains, this study attempted to use a mix of registered varieties and advanced breeding lines based on the creation timeline rather than the year of release. This was unavoidable due to the scarcity of registered varieties (especially in the earlier periods) and the resulting problems with reaching a large enough sample size. Despite this shortcoming, the GY increase over time shows similarity to many collections (Sayre et al. 1997; Abbate et al. 1998; Shearman et al. 2005; Voss-Fels et al. 2019) and our previous research on recent Norwegian varieties. However, the last three periods (1996–2015) showed considerable variance in GY and hinted to GY stagnation since 1996. This stagnation appeared due to the line composition in the last three periods: mostly advanced breeding lines and few varieties. There were no signs of GY plateau if varieties from those periods were analyzed only (Mróz et al. 2022). No trend over time was apparent for DH while a slight increase in DM and a sharp decline in PH (driven mainly by the oldest, tall accessions) could be observed. By investigating the genetic pool of the most recent wheat breeding germplasm in Norway (the validation panels), it becomes clear that favorable effect alleles of GY and DM were accumulated. This finding corresponds to Voss-Fels et al. (2019), where the authors showed that varieties gradually accumulated genetic structures associated with GY, resulting in their linear increase over the years. Interestingly, in the most recent breeding lines, alleles associated with reduced PH dominate (concerning the *Rht* loci (Pearce et al. 2011) represented by *QHt.nmbu-4B* and *QHt.nmbu-4D* in this work); however, still retaining a high degree of diversity in the loci.

From the breeding progress standpoint, the most exciting finding is the *QYld.nmbu-7B* locus. The region is strongly associated with GY, and a sharp decline in the frequency of the unfavorable allele was observed over the studied periods. One could argue that its association could be spurious and linked more to genetic background rather than GY itself—all the oldest lines carry one allele and have lower GY, in contrast to the recent accessions with other alleles and higher GY. However, the SNPs constituting this locus still show a degree of polymorphism in the validation sets, enough to be significantly associated with GY. In a nearby region (chromosome 7B, 674 Mb) to *QYld.nmbu-7B* (701 Mb), the *TaCol-5* gene was discovered and experimentally confirmed to be associated with the number of spikelet nodes per spike in wheat (Zhang et al. 2022). Transgenic plants overexpressing modified dominant *TaCol-5* allele showed increased GY due to higher tiller and spike number paired with more significant spike node number. In light of the previously mentioned GY progress in Norwegian spring wheat associated with an increase in the number of grains per spike (Mróz et al. 2022), it is compelling to hypothesize that this locus contributed to the breeding progress in GY in Norway; however, a more detailed genetic study of yield components and spike parameters is needed before endorsing this hypothesis. The exact origins of the favorable effect alleles in Norwegian germplasm remain largely unknown. However, an examination of pedigrees revealed that the high GY allele (CTAC) of *QYld.nmbu-7B* occurred for the first time in line T7347 (YOC 1977), which was a product of a cross between Runar (YOC 1965, first modern Norwegian landmark variety released in 1972) and the German variety Sirius. Since Runar carries the low GY allele (TCCT), the high GY allele must therefore come from Sirius (present in this study and indeed carries the CTAT allele). The high GY haplotype (CTAC) is also found in a group of Swedish varieties (Tjalve, Dragon, Avle, and Zebra). The other favorable allele (CTCC) is found in modern Norwegian varieties with CIMMYT parentage (Bastian, Bjarne, Bajass, and Berserk), indicating that this haplotype was introduced into Norwegian wheat breeding by crossing with CIMMYT parents. Indeed, the CTCC haplotype is present in Australian varieties with CIMMYT parents like Avocet and Kukri as well as the landmark Brazilian variety Frontana (all present in the exotic group of this study). However, despite this finding being of value for documenting breeding progress, this locus appears to be almost “fully utilized” in the most recent lines and shows no room for future improvement with the current collection of alleles present in the germplasm.

Another locus showing signs of selection in Norwegian wheat is *QYld.nmbu-3A*, which, in contrast to *QYld.nmbu-7B*, still shows a high degree of polymorphism in the most recent lines. Despite detecting only one significant SNP in the locus, its association was consistent in the validation sets. *QYld.nmbu-3A*’s only MTA appears in a similar region to the chromosomal location of the *TaGS5-3A* gene, a locus selected during breeding in Chinese wheat associated with increased kernel size, resulting in higher GY (Wang et al*.* 2015; Ma et al. 2016). No significant breeding-related progress in kernel weight was discovered in the 1972–2019 period in Norwegian spring wheat (Mróz et al. 2022); however, considering the relatively small effect of *QYld.nmbu-3A*, it could easily have been missed, had it occurred due to incorporation of this locus. *QYld.nmbu-3A* could also make a promising candidate for future improvement in GY due to the still-existing polymorphism in the most recent germplasm. To confirm *QYld.nmbu-3A*’s link with kernel weight, a follow-up study of kernel parameters in the collection is needed. The favorable allele (A) of *QYld.nmbu-3A* first appears with Møystad, an old Norwegian variety released in 1966. Møystad is a product of a cross between a sister line (Mø043-40, not present in the panel) of Norrøna (old Norwegian variety, released 1952) with the Swedish variety Kärn II. Since Norrøna carries the G allele (associated with negative effect on GY), the favorable allele (A) likely comes from Kärn II. The presence of the same allele in Swedish varieties like Dragon and Avle supports this hypothesis. The allele has been further transmitted from Møystad to important Norwegian varieties like Bastian and Bjarne, and later became dominating in the Norwegian spring wheat breeding material.

The tallness of the oldest lines investigated correlates well with the *QHt.nmbu-2A* locus, which became almost monomorphic over time with regard to the short allele. The oldest line with favorable (short) allele (ACACC) is the breeding line MS273-150 (YOC 1975) which is a progeny from the cross of Møystad with the landmark semi-dwarf variety Sonora 64. As Møystad carries the tall allele (GTCTT), it is likely that Sonora 64 was the source of the short allele. The short allele is also present in line T7347, which must have inherited it from Sirius. Moreover, the short allele is also found in Swedish varieties like Tjalve, Dragon, and Avle, which all have been used as crossing parents. Therefore, there are at least three plausible sources of the short allele in the Norwegian spring wheat breeding program. Significant MTAs with PH in a similar region were previously detected (Jamil et al. 2019), but to the authors’ knowledge, no major known gene is situated in this locus.

Reduced height (*Rht*) genes originating from the Japanese variety Norin-10 played a crucial role in the Green Revolution by introducing semi-dwarf posture to new varieties, which became spread worldwide during consecutive breeding efforts (Borojevic and Borojevic 2005). *Rht-B1* and *Rht-D1* loci are well visible in Norwegian spring wheat, represented by the *QHt.nmbu-4B* and *QHt.nmbu-4D* regions, respectively. These loci still maintain high polymorphism in Norwegian spring wheat, with slight domination of the tall alleles, indicating that also other, mostly unknown, genetic mechanisms contribute to the desired plant height of present-day varieties.

Due to the risks associated with wet periods at the end of the growing season in Norway (lodging and quality loss due to pre-harvest sprouting), early-maturing varieties of spring cereals are generally desired. However, due to climate change, it was estimated that from the 1970s until 2005, the vegetative season in Norway was extended by approximately seven days (Nordli et al. 2008). Varieties released in this period did utilize that change by extending their vegetative periods by four days on average (Mróz et al. 2022). The lack of evidence of consistent changes in allele frequencies of the discovered loci associated with DM indicates that this increase occurred due to the accumulation of several smaller-effect alleles rather than by incorporating fewer big-effect alleles. The two discovered regions associated with DM (*QMat.nmbu-6B* and *QMat.nmbu-6B*) show polymorphism and significant effects in the validation panel, with late alleles dominating the population.

## Conclusions

A detailed GWAS analysis was conducted on multi-environment field trial data of grain yield, earliness, and plant height in a diverse panel consisting of adapted and exotic lines. The study detected twelve loci associated with the traits (two with heading time, two with maturity time, three with grain yield, and five with plant height), later validated using independent sets of recent Norwegian breeding lines. The results indicated that adaptation to the Nordic growing conditions is seen mainly in changes in grain yield and is genetically associated with a phenological response due to polymorphisms in the *Vrn-A1* locus. The study also indicated that grain yield breeding progress in Norwegian spring wheat was associated with the incorporation of the *TaCol-5* and *TaGS5-3A* loci, responsible for changes in spike architecture and kernel weight; however, a detailed follow-up study on spike and kernel traits is required. The radical drop in plant height since the 1970s was associated with a locus on chromosome 2A. Knowledge of these discovered QTL regions will be useful for breeding programs targeting high-latitude spring wheat growing regions with similar growing conditions to those in Norway.

## Supplementary Information

Below is the link to the electronic supplementary material.

Supplementary file1 (XLSX 20 KB)

Supplementary file2 (XLSX 206 KB)

Supplementary file3 (XLSX 1747 KB)

Supplementary file4 (XLSX 160 KB)

Supplementary file5 (DOCX 10414 KB)

## Data Availability

Data and material available upon reasonable request to the corresponding author.

## Abbreviations

Chr: Chromosome
CIMMYT: International maize and wheat improvement center
CMLM: Compressed mixed linear model
DArT: Diversity array technology
DH: Days to heading
DM: Days to maturity
FarmCPU: Fixed and random model circulating probability unification
FDR: False discovery rate
gBLUP: Genomic best linear unbiased predictions
GP: Genomic prediction
GWAS: Genome-wide association study
GY: Grain yield
HSD: Honestly significant difference
LD: Linkage disequilibrium
MAS: Marker-assisted selection
MLM: Mixed linear model
MLMM: Multi-locus mixed model
MQTL: Meta-QTL analysis
MTA: Marker-trait association
PCA: Principal component analysis
PH: Plant height
QQ-plot: Quantile–quantile plot
QTL: Quantitative trait locus
QTN: Quantitative trait nucleotide
SNP: Single nucleotide polymorphism
SSR: Simple sequence repeats
SUPER: Settlement of MLM under progressively exclusive relationship
