# Supplementary material for: Grain yield and adaptation of spring wheat to Norwegian growing conditions is driven by allele frequency changes at key adaptive loci discovered by genome-wide association mapping
Source: Theor Appl Genet. 2023 Aug 17;136(9):191. doi: 10.1007/s00122-023-04424-9 (PMC10435424; doi:10.1007/s00122-023-04424-9)
Supplement: Supplementary file 5 — Supplementary file5 (DOCX 10414 KB) [file 122_2023_4424_MOESM5_ESM.docx]

**Supplementary information**

**Grain yield and adaptation of spring wheat to Norwegian growing conditions is driven by allele frequency changes at key adaptive loci discovered by genome-wide association mapping**

Tomasz Mroz^1^, Jon Arne Dieseth^2^ & Morten Lillemo^1^

^1^Department of Plant Sciences, Norwegian University of Life Sciences, NO-1432 Ås, Norway

^2^Graminor, AS, Bjørke Gård, Hommelstadvegen 60, NO‐2322 Ridabu, Norway

Corresponding author:

Morten Lillemo

morten.lillemo@nmbu.no

Orcid-ID: 0000-0002-8594-8794

**Table S1** Planting dates for the main and validation panels’ trials

| Season | Vollebekk | | Staur | |
| --- | --- | --- | --- | --- |
|  | **Main panel** | **Validation panel** | **Main panel** | **Validation panel** |
| 2015 | 2015-04-24 | - | - | - |
| 2016 | 2016-04-24 | - | 2016-05-10 | - |
| 2017 | 2017-05-04 | - | 2017-05-12 | - |
| 2018 | 2018-05-10 | - | 2018-05-09 | - |
| 2019 | 2019-05-19 | 2019-04-24 | 2019-06-04 | - |
| 2020 | 2020-05-15 | 2020-04-15 | 2020-04-21 | 2020-04-21 |
| 2021 | 2021-04-20 | 2021-04-19 | 2021-04-27 | 2021-04-27 |
| 2022 | - | 2022-04-26 | - | - |

**Table S2** Overview of the number of lines which were phenotyped in each season/environment combination in the main panel

| Location | Field season | | | | | | |
| --- | --- | --- | --- | --- | --- | --- | --- |
|  | **2015** | **2016** | **2017** | **2019** | **2020** | **2021** | **Total†** |
| Vollebekk | 163 | 100 | 240 | 220 | 288 | 295 | 301 |
| Staur | - | 100 | 240 | 220 | 288 | - | 296 |

**†** Total number of unique lines for which a cross-season, cross-environment means were computed

**Table S3** Overview of the number of lines phenotyped in each season/location combination in the validation panels

| Location | Field season | | | | Total unique lines |
| --- | --- | --- | --- | --- | --- |
|  | **2019** | **2020** | **2021** | **2022** |  |
| Vollebekk | 309 | 397 | 267 | 265 | 889 |
| Staur | 90 | 354 | - | - |  |


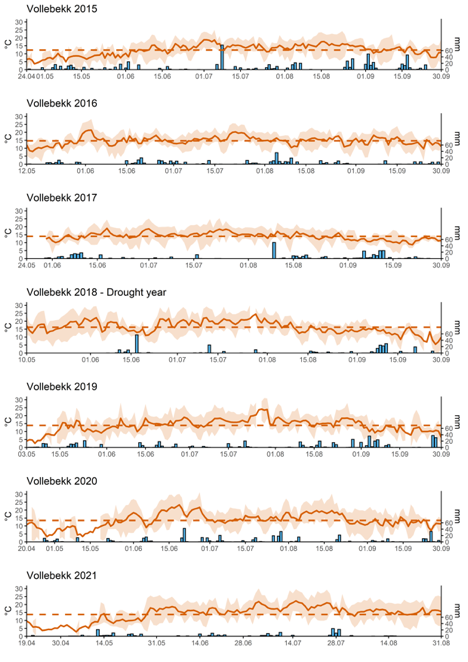


**Fig. S1** Weather data for field seasons 2015-2021 in Vollebekk research station, from seeding the trial to the end of September. Solid line marks the average temperature for each day, dashed line marks the mean temperature across the season, bars mark the daily rainfall in mm per m^2^. Primary y axis – temperature in ⁰C, secondary y axis – rainfall in mm per m^2^


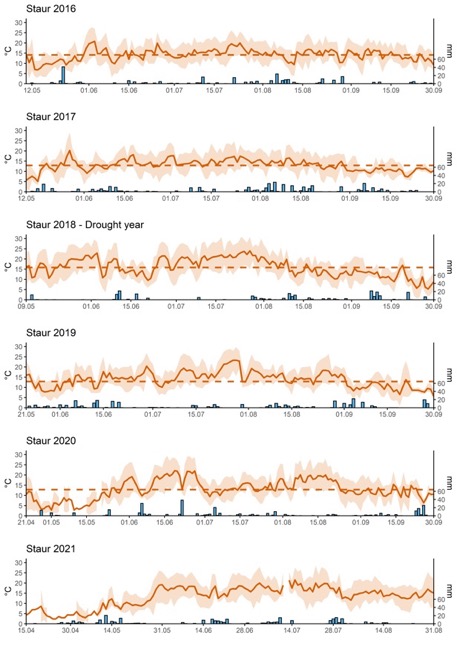


**Fig. S2** Weather data for field seasons 2016-2021 in Staur, from seeding the trial to the end of September. Solid line marks the average temperature for each day, dashed line marks the mean temperature across the season, bars mark the daily rainfall in mm per m^2^. Primary y axis – temperature in ⁰C, secondary y axis – rainfall in mm per m^2^

**Table S4** Descriptive statistics of weather conditions at Vollebekk research station over the analyzed growing seasons. T_avg_ – average temperature in °C, Rf_sum_ – sum of rainfall in mm, Ir_avg_ – average solar irradiance in Wm^-2^

|  | 2015 | | | 2016 | | | 2017 | | | 2018 (drought) | | | 2019 | | | 2020 | | | 2021 | | |
| --- | --- | --- | --- | --- | --- | --- | --- | --- | --- | --- | --- | --- | --- | --- | --- | --- | --- | --- | --- | --- | --- |
|  | T_avg_ | Rf_sum_ | Ir_avg_ | T_avg_ | Rf_sum_ | Ir_avg_ | T_avg_ | Rf_sum_ | Ir_avg_ | T_avg_ | Rf_sum_ | Ir_avg_ | T_avg_ | Rf_sum_ | Ir_avg_ | T_avg_ | Rf_sum_ | Ir_avg_ | T_avg_ | Rf_sum_ | Ir_avg_ |
| Apr | 5.5 | 8.8 | 15.4 | 5.2 | 100 | 11.7 | 4.4 | 35.0 | 13.4 | 5.1 | 32.4 | 14,0 | 7.9 | 14.6 | 16.2 | 6.4 | 30.2 | 15.1 | 4.7 | 18.2 | 17.1 |
| May | 8.3 | 116.6 | 16.3 | 11.6 | 50.2 | 18.5 | NA | 68.0 | NA | 15.0 | 0 | 22.2 | 9.7 | 100.6 | 17.0 | 9.5 | 47.2 | 21.4 | 9.7 | 72.2 | 15.4 |
| Jun | 13.1 | 65.6 | 21.4 | 15.6 | 89.8 | 21.1 | 14.3 | 94.0 | 19.1 | 16.7 | 82.4 | 23.9 | 14.8 | 63.6 | 17.8 | 17.6 | 115.4 | 21.2 | 16.1 | 34.6 | 19.9 |
| Jul | 15.0 | 158.8 | 19.4 | 16.1 | 54.8 | 19.1 | 15.9 | 20.2 | 19.8 | 20.2 | 44.6 | 23.2 | 17.2 | 52.0 | 20.2 | 14.3 | 127.9 | 18.0 | 18.9 | 95.4 | 19.8 |
| Aug | 15.3 | 140.2 | 16.0 | 14.6 | 140 | 14.1 | 14.5 | 104.0 | 14.9 | 15.4 | 21.4 | 14.2 | 16.2 | 110.2 | 13.7 | 16.2 | 50.6 | 15.7 | 15.3 | 7.8 | 15.2 |
| Sep | 11.5 | 220.2 | 8.8 | 14.1 | 41 | 9.9 | 11.5 | 119.2 | 6.7 | 12.1 | 128.4 | 9.4 | 11.0 | 190.8 | 8.3 | 12.0 | 81.0 | 9.0 | 12.5 | 75.2 | 8.7 |

**Table S5** Descriptive statistics of weather conditions at Staur research farm over the analyzed growing seasons. T_avg_ – average temperature in °C, Rf_sum_ – sum of rainfall in mm, Ir_avg_ – average solar irradiance in Wm^-2^

|  | 2015 | | | 2016 | | | 2017 | | | 2018 (Drought) | | | 2019 | | | 2020 | | | 2021 | | |
| --- | --- | --- | --- | --- | --- | --- | --- | --- | --- | --- | --- | --- | --- | --- | --- | --- | --- | --- | --- | --- | --- |
|  | T_avg_ | R_fsum_ | Ir_avg_ | T_avg_ | R_fsum_ | Ir_avg_ | T_avg_ | R_fsum_ | Ir_avg_ | T_avg_ | R_fsum_ | Ir_avg_ | T_avg_ | R_fsum_ | Ir_avg_ | T_avg_ | R_fsum_ | Ir_avg_ | T_avg_ | R_fsum_ | Ir_avg_ |
| Apr | 4.6 | 6.9 | 14.0 | 3.9 | 74.3 | 12.1 | 3.6 | 33.4 | 12.9 | 3.5 | 40.3 | 13.8 | 5.6 | 3.0 | 15.3 | 5.5 | 19.4 | 14.3 | 3.7 | 16.0 | 15.5 |
| May | 7.4 | 89.0 | 14.2 | 10.6 | 66.5 | 16.8 | 10.1 | 59.3 | 15.7 | 15.1 | 22.8 | 21.1 | 8.5 | 91.4 | 15.1 | 8.3 | 33.8 | 18.5 | 9.0 | 81.2 | 14.9 |
| Jun | 12.6 | 39.3 | 19.9 | 15.2 | 29.5 | 20.7 | 13.5 | 58.3 | 17.3 | 16.0 | 55.8 | 22.3 | 14.4 | 103.6 | 16.8 | 17.4 | 98.9 | 21.5 | 16.4 | 62.4 | 20.7 |
| Jul | 14.8 | 116.3 | 16.4 | 15.8 | 59.4 | 17.7 | 15.0 | 58.8 | 18.3 | 20.7 | 24.8 | 22.4 | 16.6 | 37.6 | 19.6 | 13.3 | 70.8 | 16.8 | NA | 93.4 | NA |
| Aug | 14.6 | 52.7 | 15.8 | 14.2 | 109.1 | 12.9 | 13.7 | 144.2 | 13.9 | 14.7 | 61.2 | 13.6 | 15.5 | 63.1 | 12.4 | 16.0 | 17.1 | 15.7 | 14.3 | 16.4 | 14.2 |
| Sep | 10.9 | 164.6 | 7.3 | 13.6 | 21.4 | 9.4 | 10.3 | 64.3 | 5.4 | 11.1 | 85.1 | 8.6 | 9.9 | 103.2 | 7.2 | 11.3 | 80.7 | 8.1 | 11.6 | 34.1 | 7.8 |


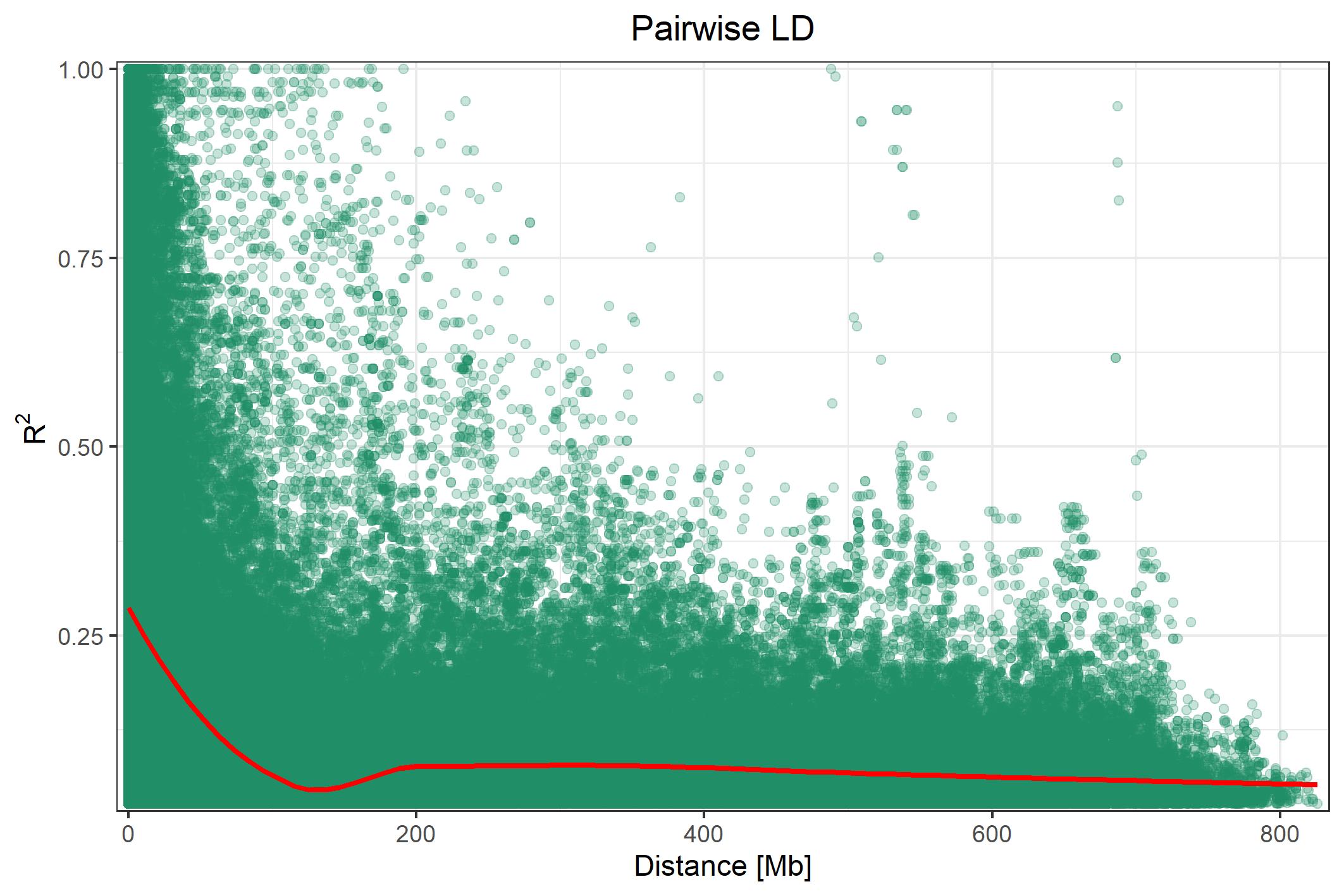


**Figure S3** Linkage disequilibrium (LD) between markers for the main panel

**Figure S4** Pearson’s correlation matrix for days to maturity between trial means, environmental means, and the global mean. Variables named according to scheme: Location_season. V – Vollebekk, S – Staur

**Figure S5** Pearson’s correlation matrix for grain yield between trial means, environmental means, and the global mean. Variables named according to scheme: Location_season. V – Vollebekk, S – Staur

**Figure S6** Pearson’s correlation matrix for days to heading between trial means, environmental means, and the global mean. Variables named according to scheme: Location_season. V – Vollebekk, S – Staur

**Figure S7** Pearson’s correlation matrix for plant height between trial means, environmental means, and the global mean. Variables named according to scheme: Location_season. V – Vollebekk, S – Staur


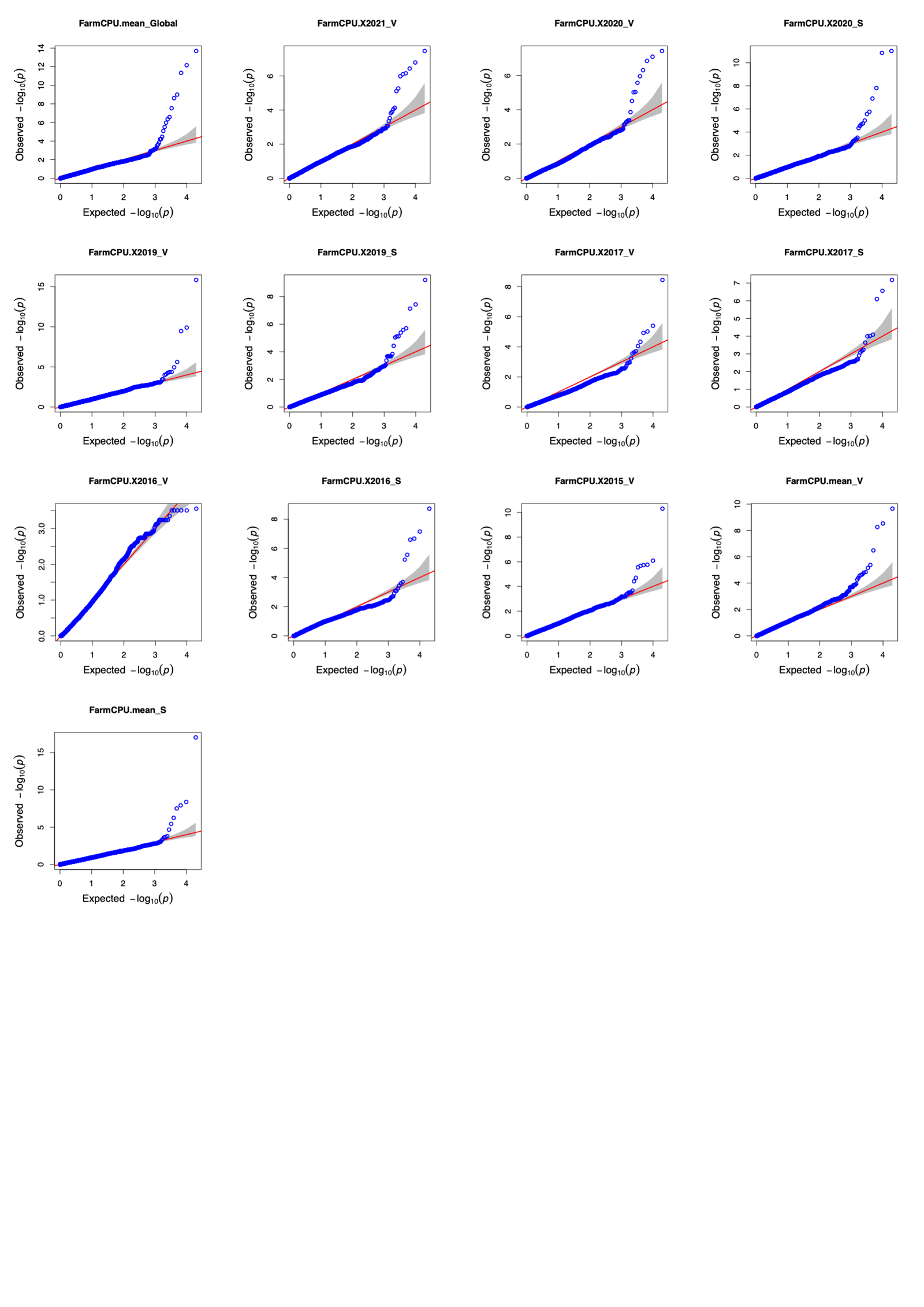


**Figure S8** Quantile-quantile (QQ) plots for GWAS analysis of days to maturity for the adapted part of the main panel. Phenotypes named according to the scheme: FarmCPU.season_location. Location: V – Vollebekk, S – Staur


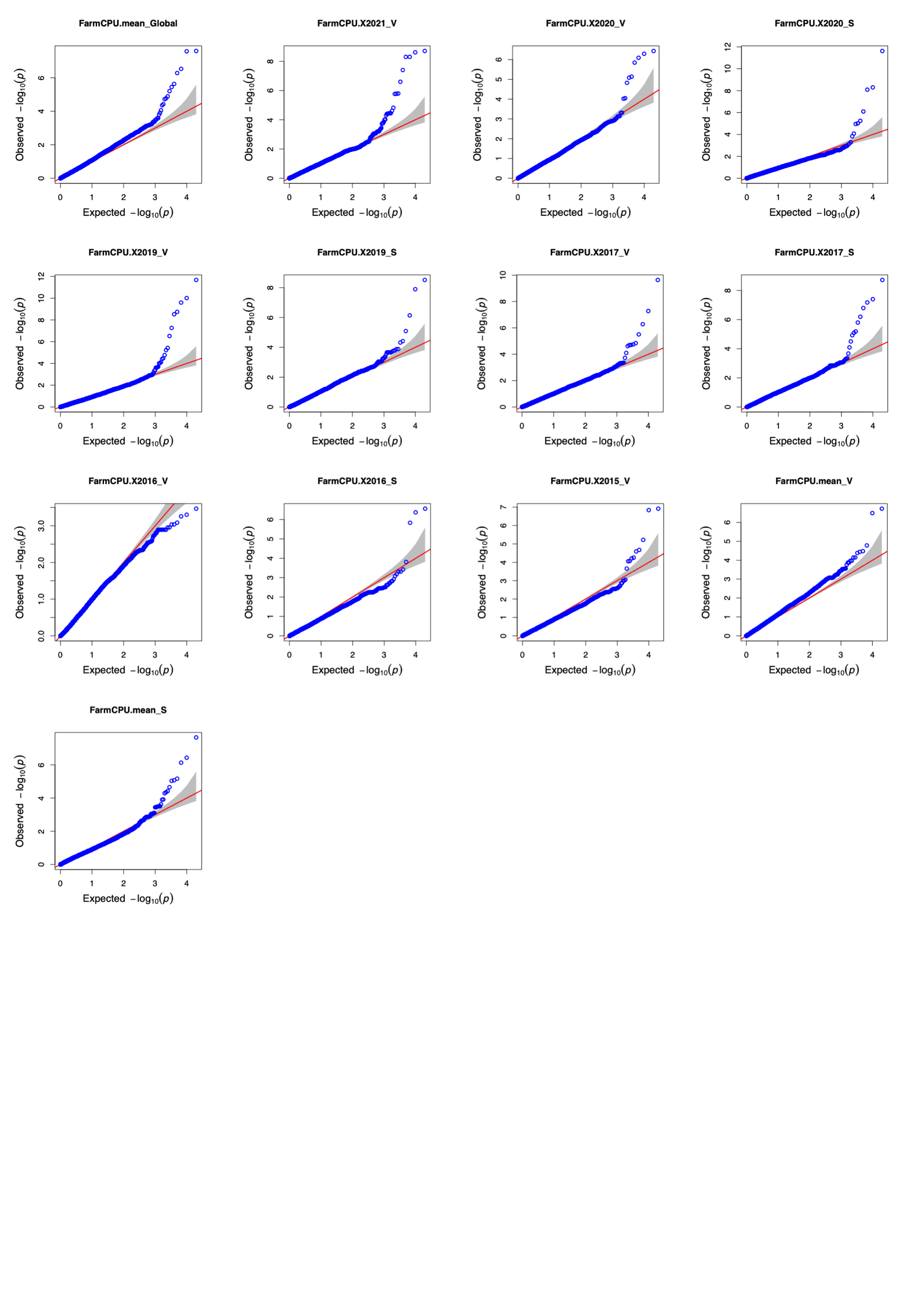


**Figure S9** Quantile-quantile (QQ) plots for GWAS analysis of days to maturity for the main panel (all lines). Phenotypes named according to the scheme: FarmCPU.season_location. Location: V – Vollebekk, S – Staur


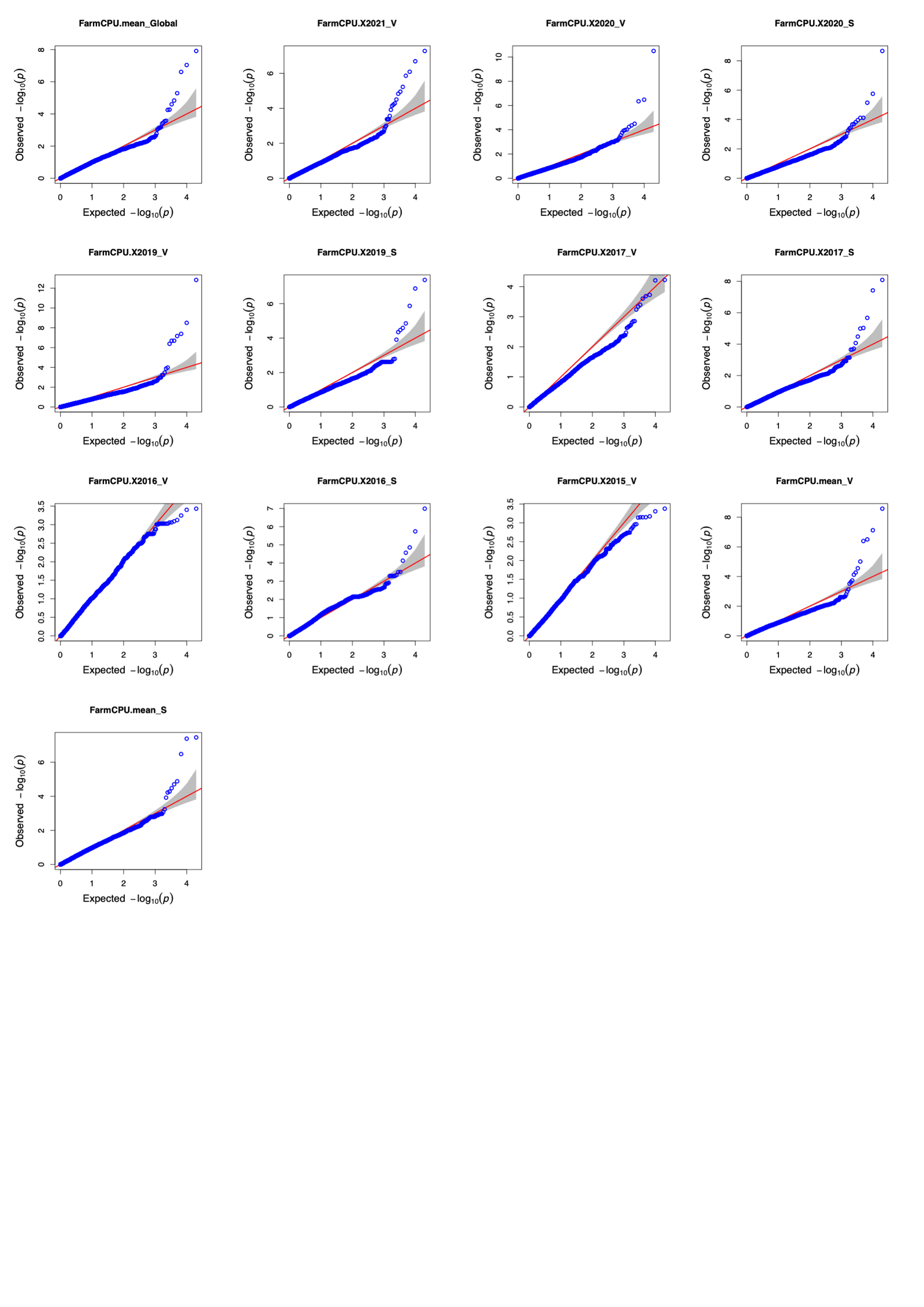


**Figure S10** Quantile-quantile (QQ) plots for GWAS analysis of grain yield for the adapted part of the main panel. Phenotypes named according to the scheme: FarmCPU.season_location. Location: V – Vollebekk, S – Staur


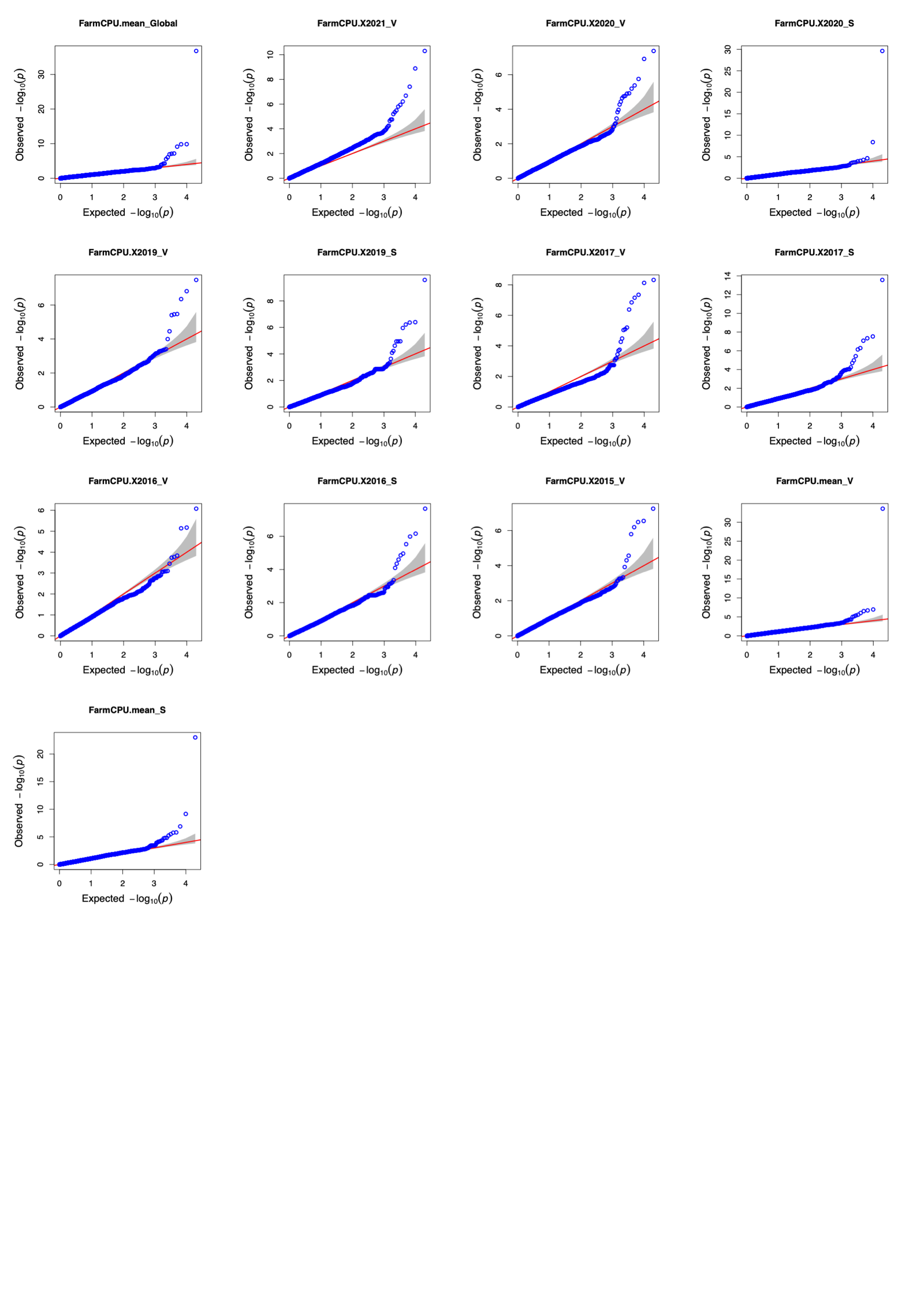


**Figure S11** Quantile-quantile (QQ) plots for GWAS analysis of grain yield for the main panel (all lines). Phenotypes named according to the scheme: FarmCPU.season_location. Location: V – Vollebekk, S – Staur.


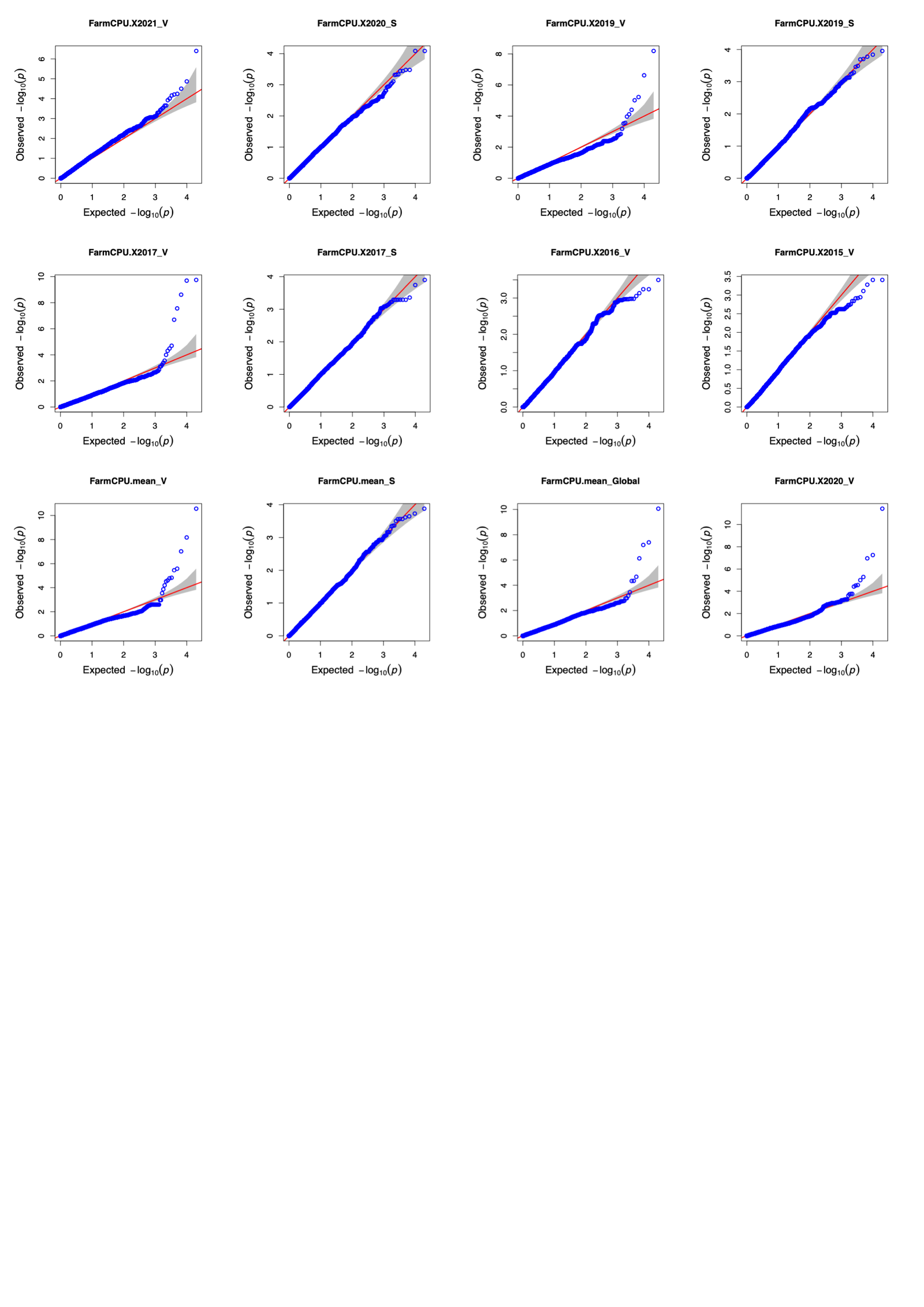


**Figure S12** Quantile-quantile (QQ) plots for GWAS analysis of days to heading for the adapted part of the main panel. Phenotypes named according to the scheme: FarmCPU.season_location. Location: V – Vollebekk, S – Staur


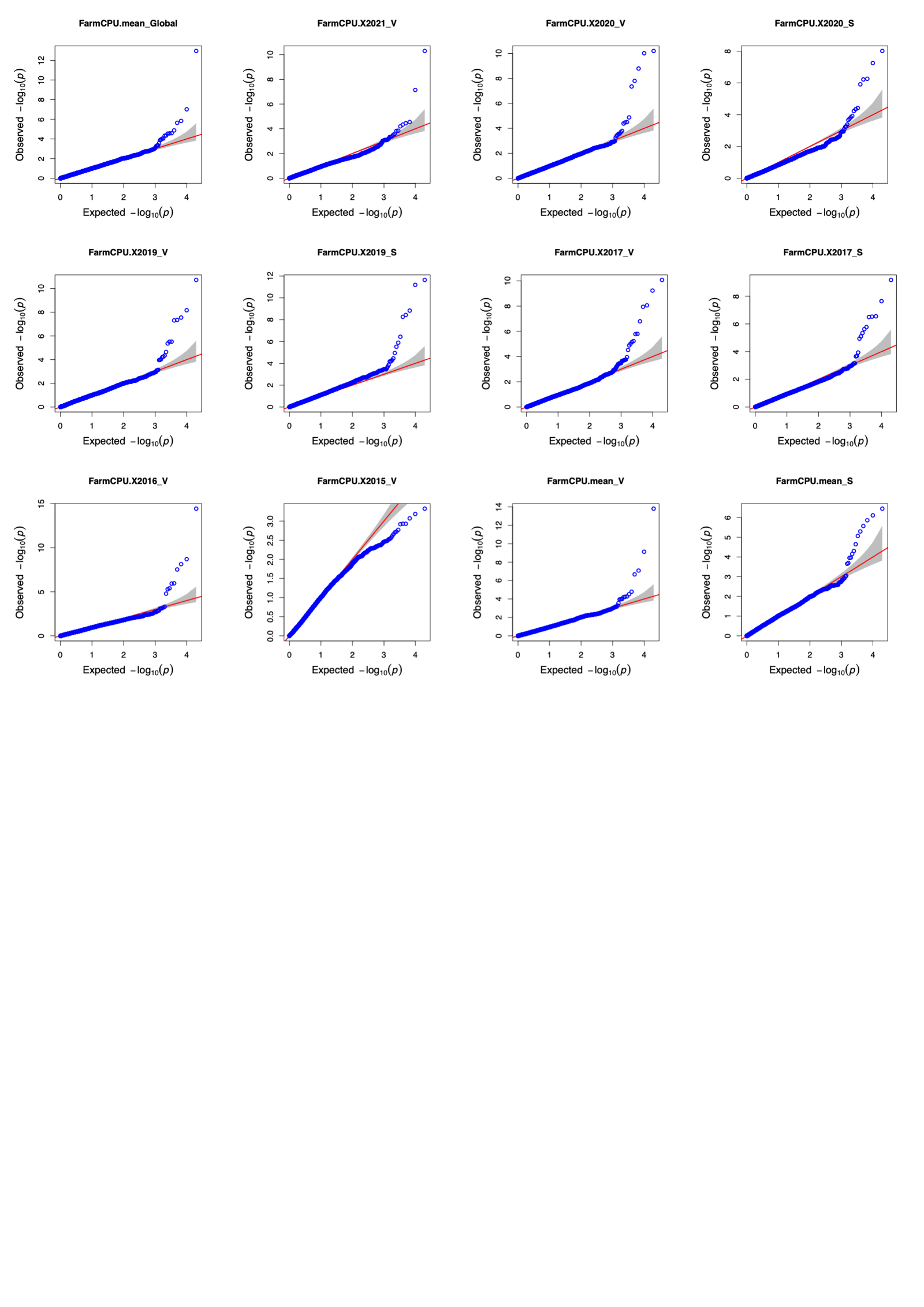


**Figure S13** Quantile-quantile (QQ) plots for GWAS analysis of days to heading for the main panel (all lines). Phenotypes named according to the scheme: FarmCPU.season_location. Location: V – Vollebekk, S – Staur


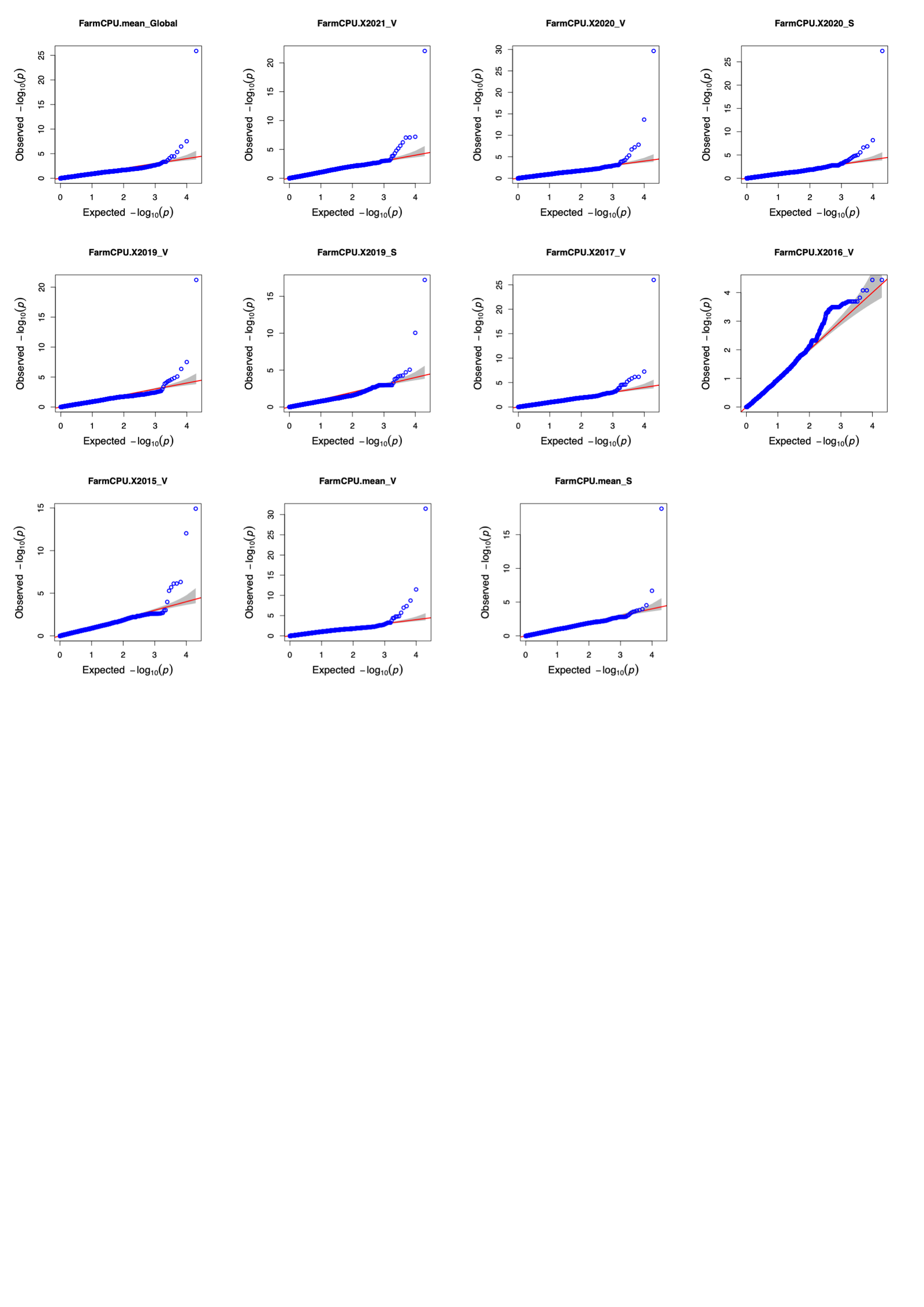


**Figure S14** Quantile-quantile (QQ) plots for GWAS analysis of plant height for the adapted part of the main panel. Phenotypes named according to the scheme: FarmCPU.season_location. Location: V – Vollebekk, S – Staur


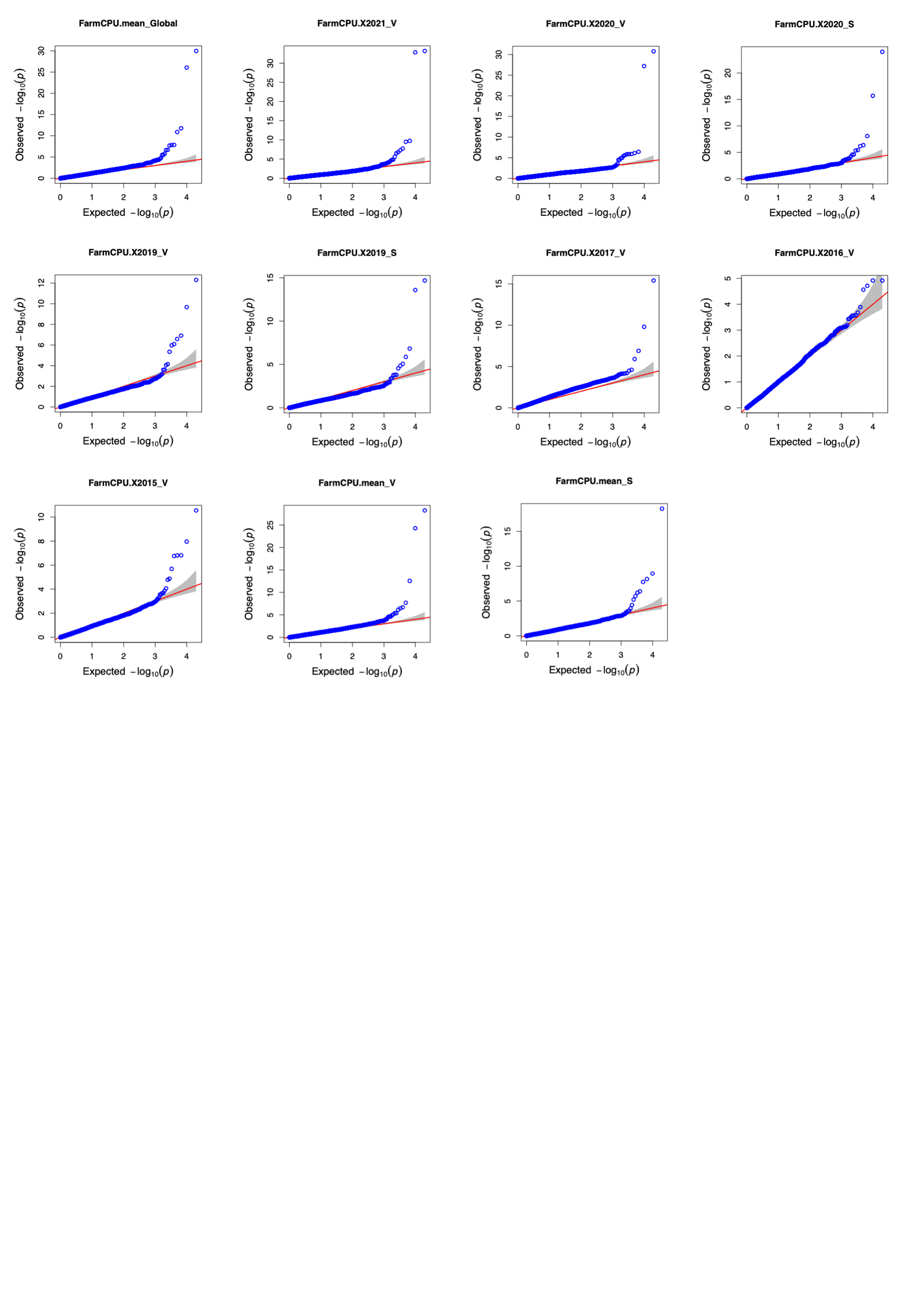


**Figure S15** Quantile-quantile (QQ) plots for GWAS analysis of plant height for the main panel (all lines). Phenotypes named according to the scheme: FarmCPU.season_location. Location: V – Vollebekk, S – Staur


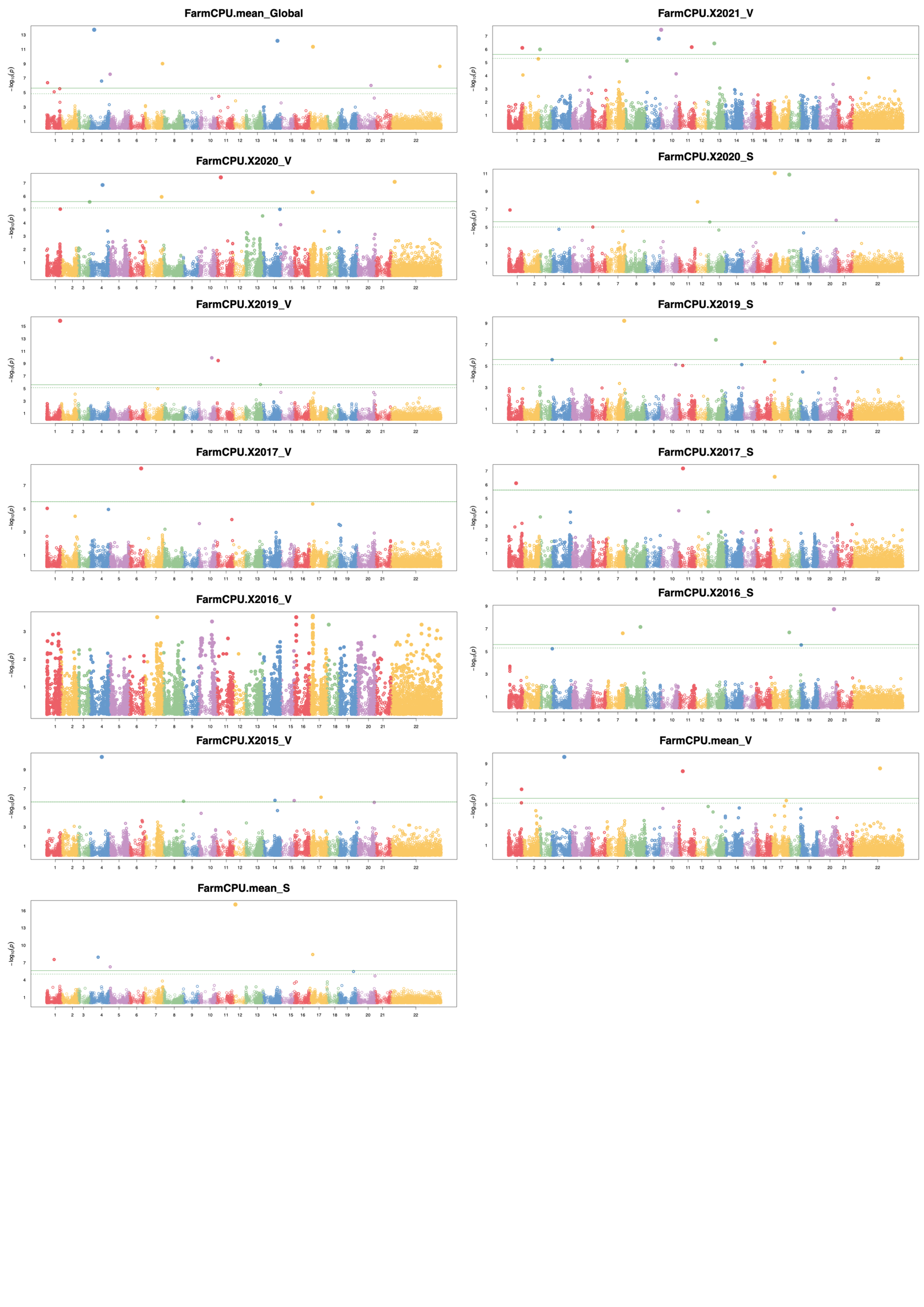


**Figure S16** Manhattan plots for GWAS analysis of days to maturity for the adapted part of the main panel. Phenotypes named according to the scheme: FarmCPU.season_location. Location: V – Vollebekk, S – Staur


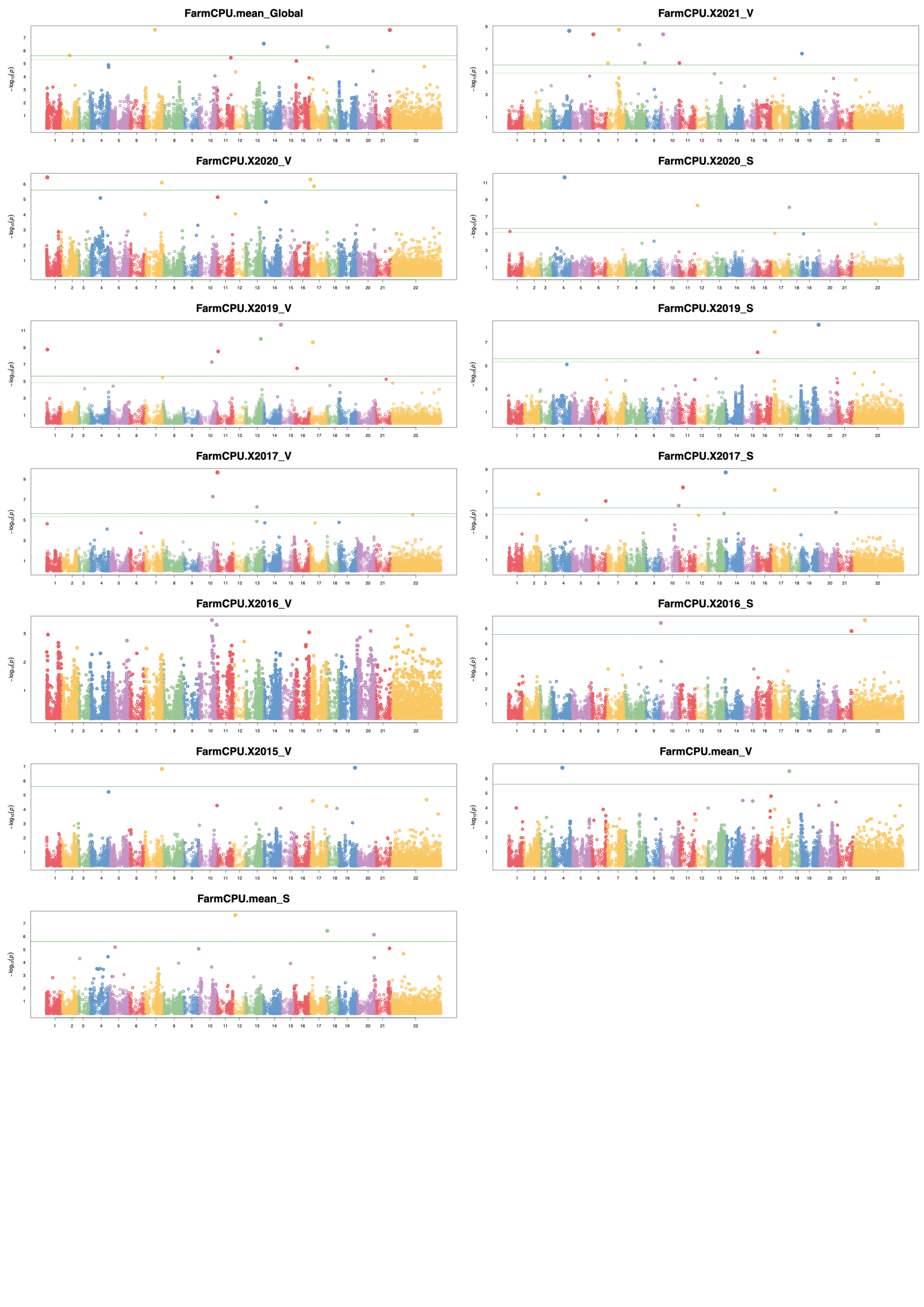


**Figure S17** Manhattan plots for GWAS analysis of days to maturity for the main panel (all lines). Phenotypes named according to the scheme: FarmCPU.season_location. Location: V – Vollebekk, S – Staur


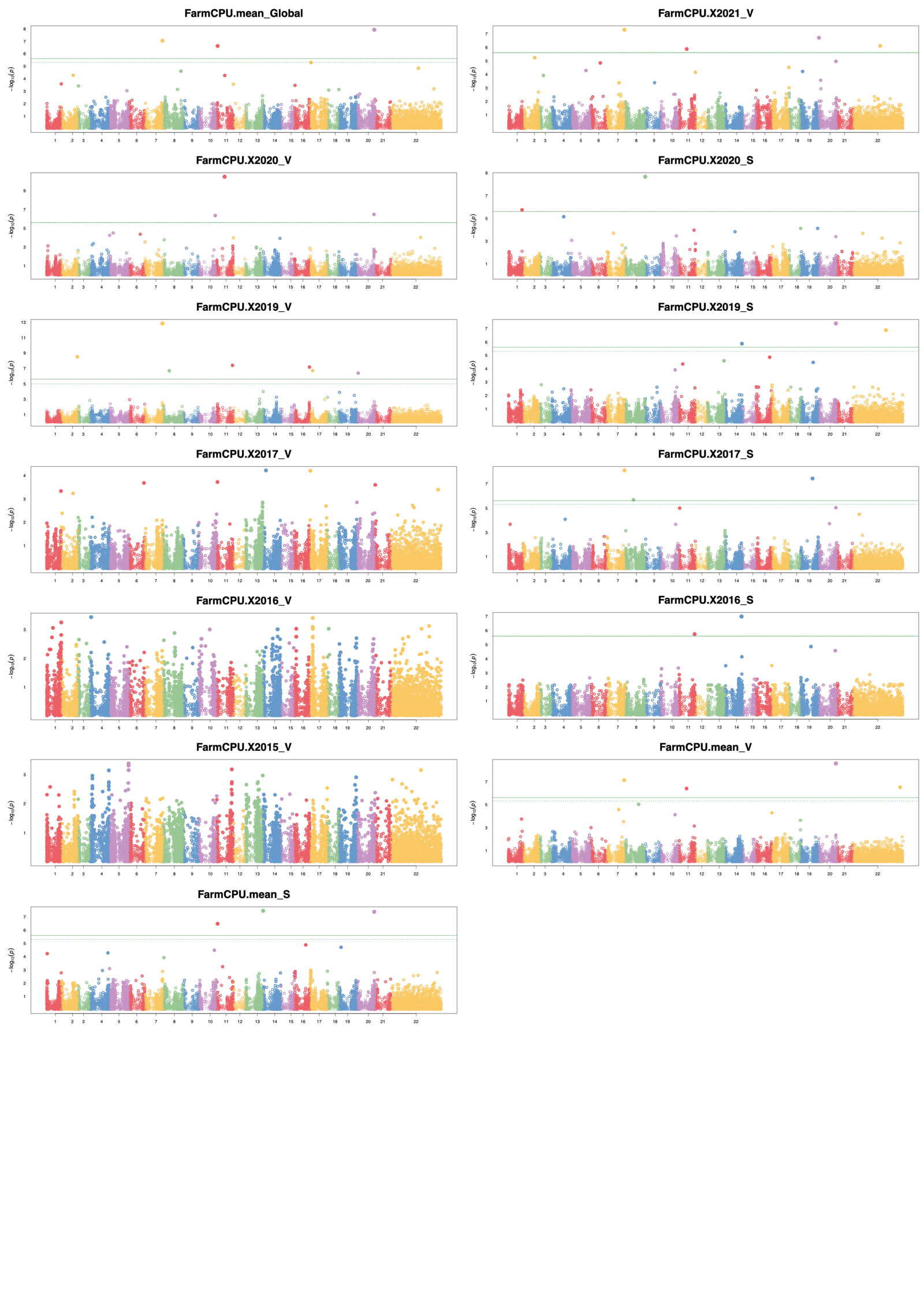


**Figure S18** Manhattan plots for GWAS analysis of grain yield for the adapted part of the main panel. Phenotypes named according to the scheme: FarmCPU.season_location. Location: V – Vollebekk, S – Staur


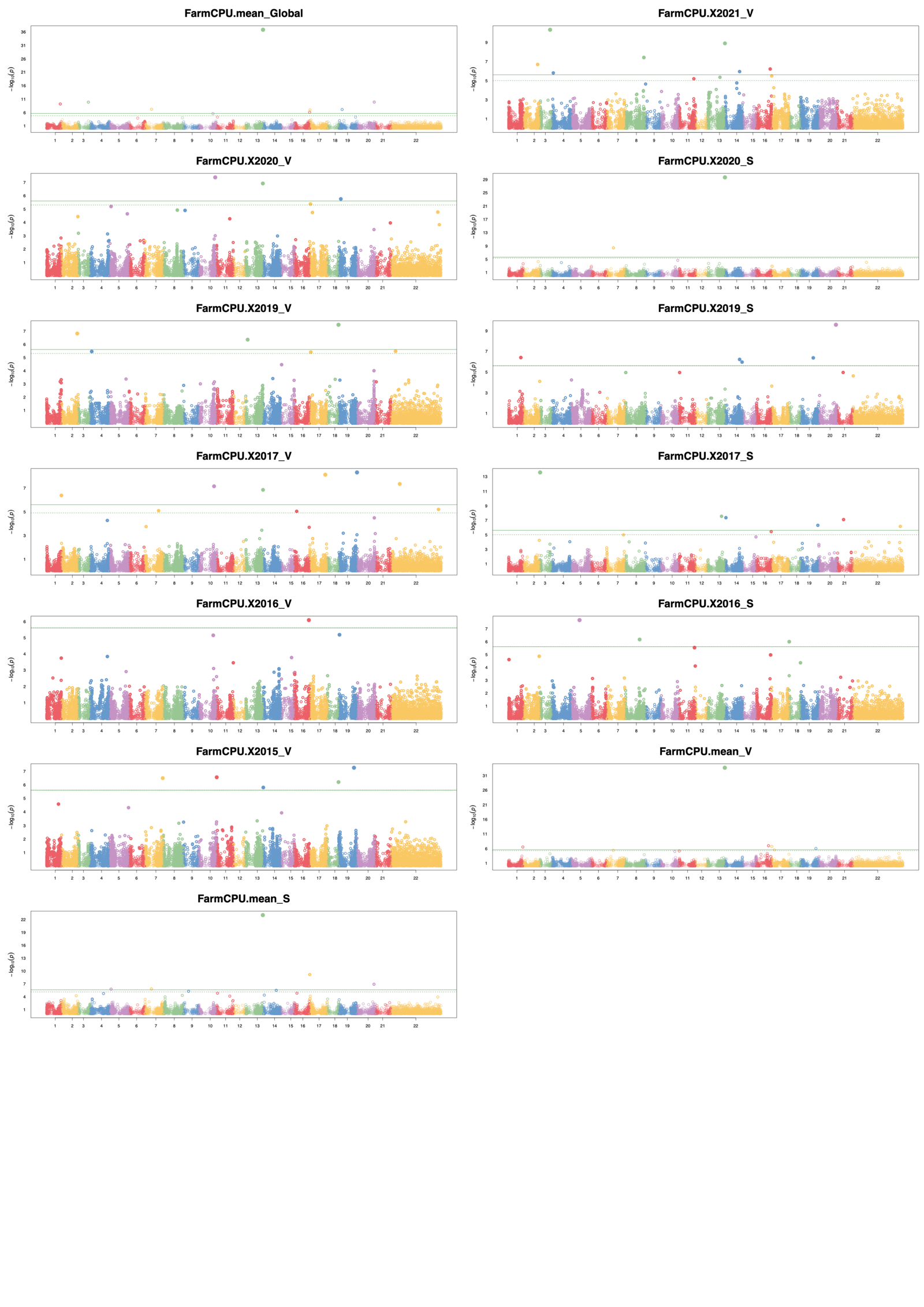


**Figure S19** Manhattan plots for GWAS analysis of grain yield for the main panel (all lines). Phenotypes named according to the scheme: FarmCPU.season_location. Location: V – Vollebekk, S – Staur


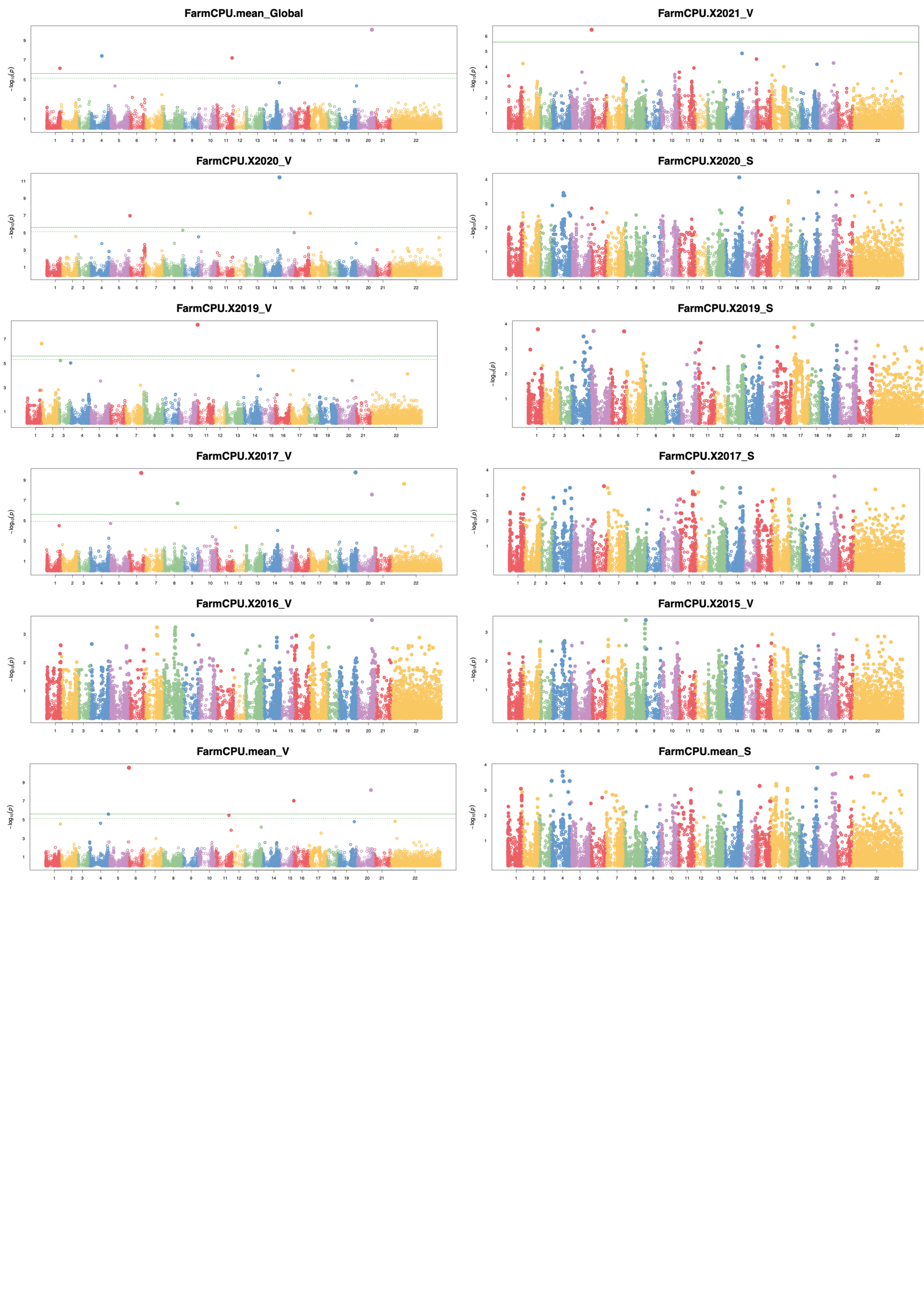


**Figure S20** Manhattan plots for GWAS analysis of days to heading for the adapted part of the main panel. Phenotypes named according to the scheme: FarmCPU.season_location. Location: V – Vollebekk, S – Staur


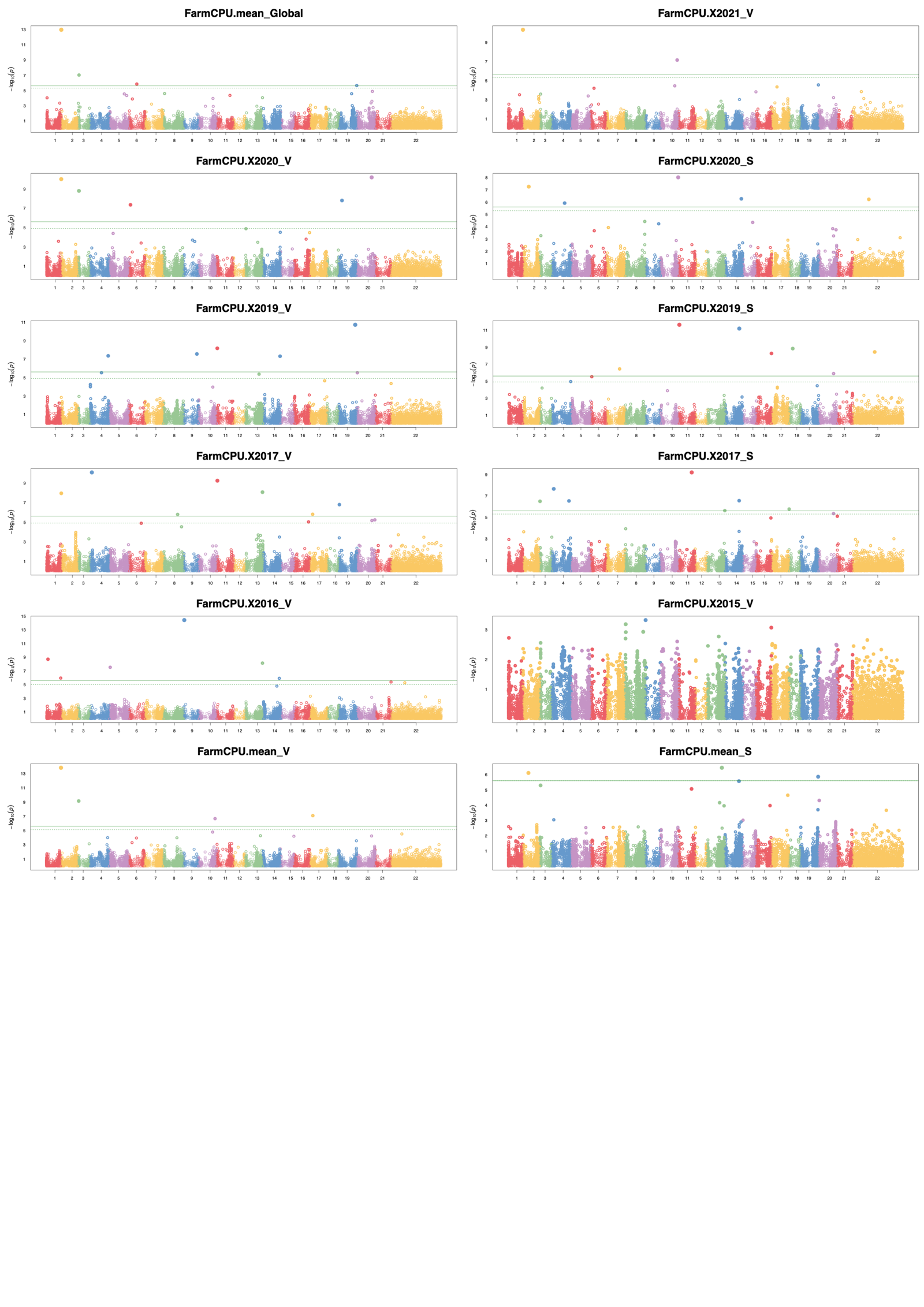


**Figure S21** Manhattan plots for GWAS analysis of days to heading for the main panel (all lines). Phenotypes named according to the scheme: FarmCPU.season_location. Location: V – Vollebekk, S – Staur


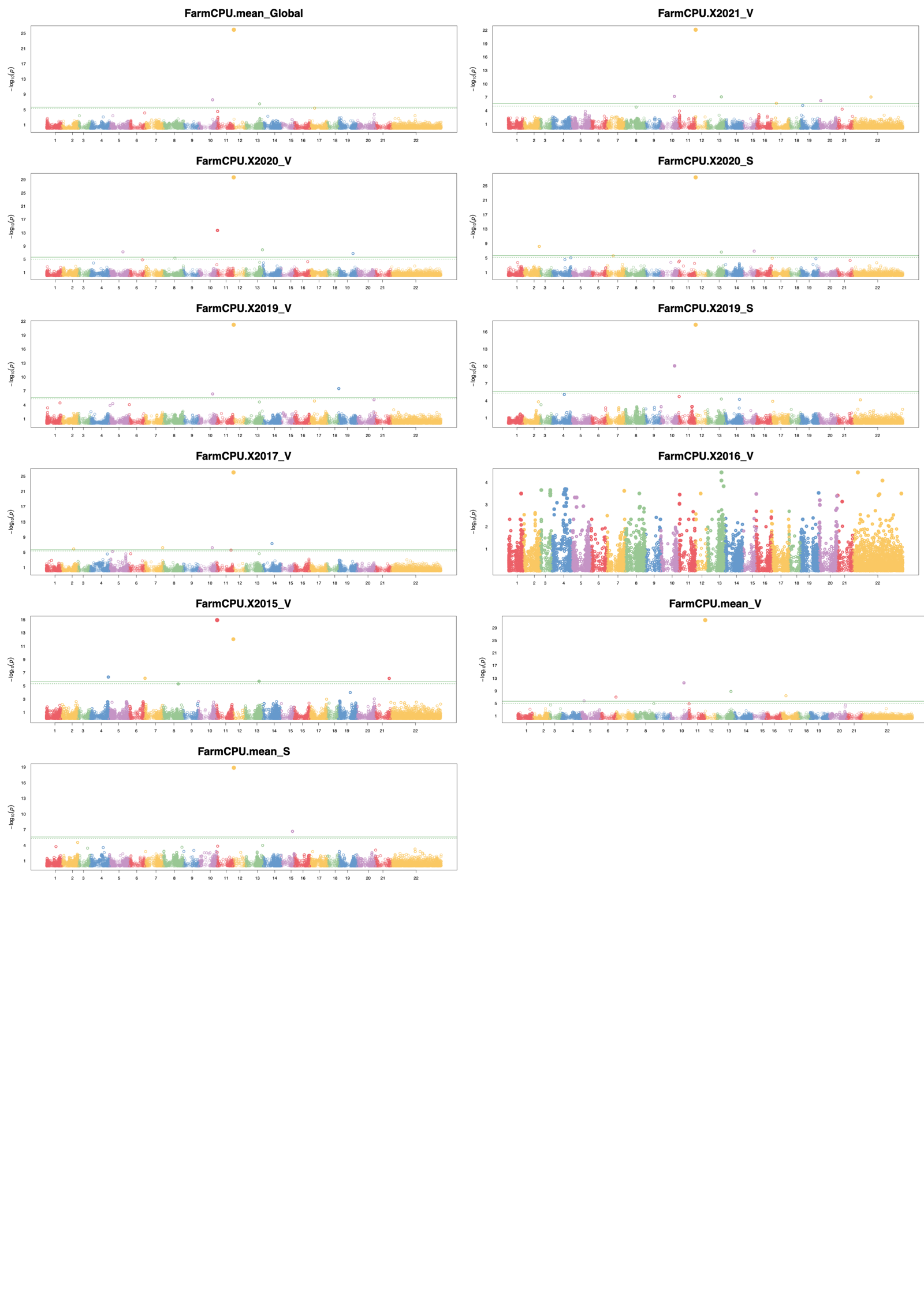


**Figure S22** Manhattan plots for GWAS analysis of plant height for the adapted part of the main panel. Phenotypes named according to the scheme: FarmCPU.season_location. Location: V – Vollebekk, S – Staur


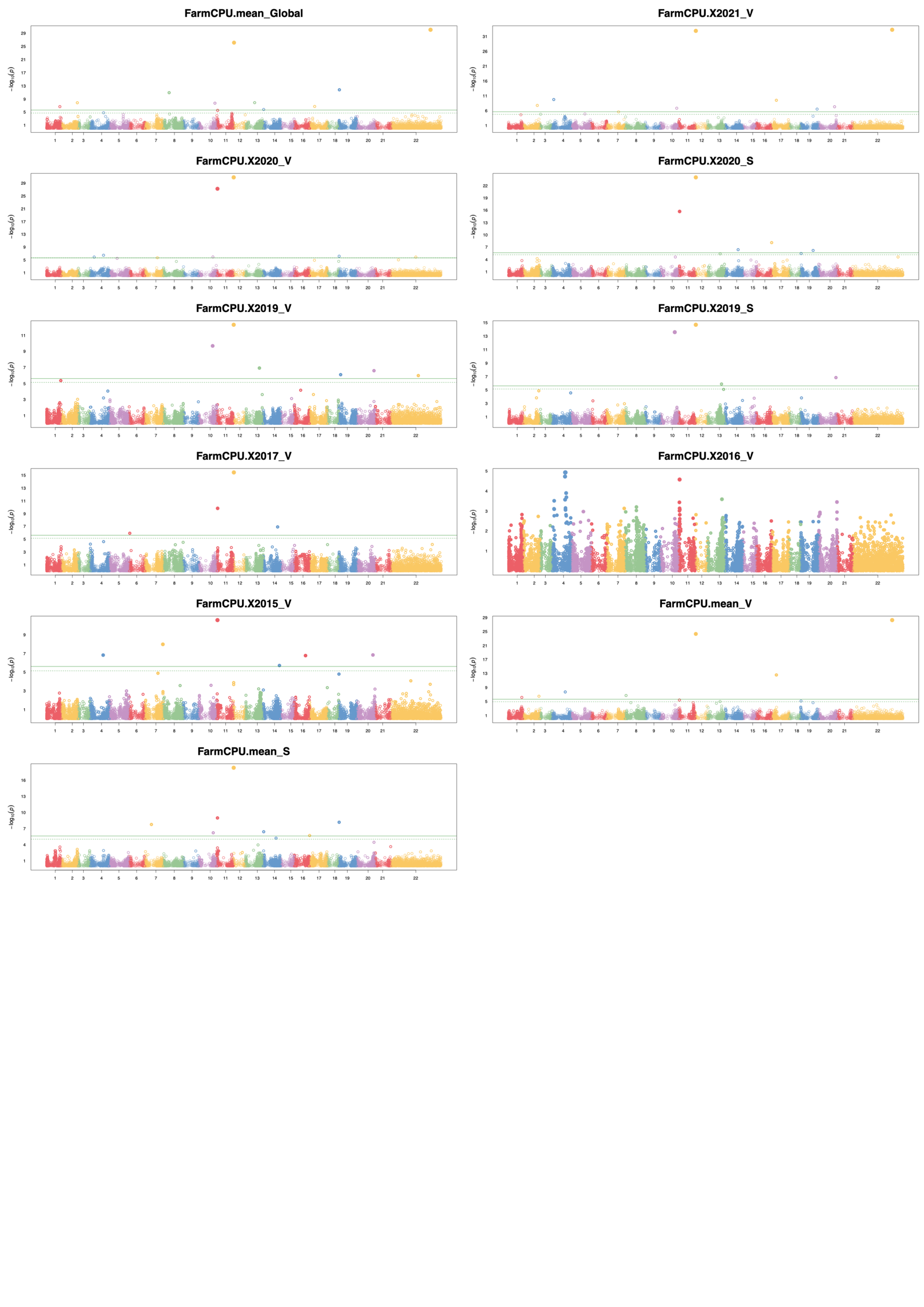


**Figure S23** Manhattan plots for GWAS analysis of plant height for the main panel (all lines). Phenotypes named according to the scheme: FarmCPU.season_location. Location: V – Vollebekk, S – Staur

**Table S6** Significant markers belonging to the discovered QTL regions for days to heading (DH), days to maturity (DM), grain yield (GY), and plant height (PH). Pos – SNP position in Mbp, E – effect (without correcting for population structure) in the full dataset, %PVE – percentage of variance explained in the full dataset, without correcting for population structure, MAF – minor allele frequency

| Trait | QTL | Span | Marker | Pos | -logP | Dataset | Method | E | %PVE | MAF |
| --- | --- | --- | --- | --- | --- | --- | --- | --- | --- | --- |
| DH | ***QHd.nmbu-1B*** | **1-2** | **BS00022180_51** | 1 | 12.95 | Full | FarmCPU, MLM | 1.02 | 14.0 | 0.08 |
|  |  |  | BS00071161_51 | 2 | 3.65 | Full/Adapted | MLM | 0.95 | 13.3 | 0.08 |
|  | ***QHd.nmbu-7B*** | **606** | **BobWhite_c3541_152** | 606 | 10.06 | Adapted | FarmCPU, MLM | 0.91 | 11.4 | 0.06 |
| DM | ***QMat.nmbu-6B*** | **132-136** | wsnp_Ex_rep_c102159_87386822 | 131 | 4.22 | Full/Adapted | MLM | 0.63 | 3.51 | 0.21 |
|  |  |  | **Kukri_rep_c71420_511** | 132 | 11.34 | Adapted | FarmCPU, MLM | 1.36 | 14.2 | 0.14 |
|  |  |  | Tdurum_contig14559_741 | 132 | 5.73 | Full/Adapted | MLM | 0.61 | 4.12 | 0.28 |
|  |  |  | Tdurum_contig14559_78 | 132 | 4.78 | Full/Adapted | MLM | 0.60 | 4.05 | 0.28 |
|  |  |  | wsnp_Ex_c12577_20022294 | 132 | 3.16 | Full/Adapted | MLM | 0.28 | 0.92 | 0.32 |
|  |  |  | GENE-3171_203 | 135 | 3.69 | Adapted | MLM | 0.97 | 9.74 | 0.21 |
|  |  |  | GENE-4074_537 | 135 | 4.22 | Full/Adapted | MLM | 0.67 | 3.57 | 0.17 |
|  |  |  | AX-158529874 | 136 | 3.11 | Adapted | MLM | 0.89 | 8.55 | 0.21 |
|  |  |  | AX-158552731 | 136 | 3.50 | Adapted | MLM | 1.06 | 10.4 | 0.18 |
|  |  |  | Tdurum_contig11700_1247 | 136 | 3.11 | Adapted | MLM | 0.89 | 8.55 | 0.21 |
|  | ***QMat.nmbu-6D*** | **6** | **BS00022523_51** | 6 | 6.28 | Full | FarmCPU, MLM | 0.82 | 8.80 | 0.41 |
| GY | ***QYld.nmbu-3A*** | **267** | **BS00110129_51** | 267 | 7.16 | Full | FarmCPU, MLM | 38.1 | 22.0 | 0.42 |
|  | ***QYld.nmbu-5A*** | **683-708** | AX-158558760 | 685 | 3.50 | Full | MLM | 37.5 | 17.5 | 0.27 |
|  |  |  | AX-94387378 | 686 | 3.17 | Full | MLM | 42.2 | 24.0 | 0.31 |
|  |  |  | AX-95097524 | 694 | 3.82 | Full | MLM | 52.6 | 34.6 | 0.28 |
|  |  |  | **BobWhite_c8266_227** | 699 | 36.78 | Full | FarmCPU, MLM | 79.4 | 61.5 | 0.20 |
|  |  |  | AX-158550750 | 708 | 3.55 | Full | MLM | 59.2 | 41.3 | 0.25 |
|  |  |  | wsnp_Ex_c2171_4072774 | 708 | 3.47 | Full | MLM | 59.3 | 41.3 | 0.25 |
|  |  |  | wsnp_Ex_c2171_4072995 | 708 | 3.47 | Full | MLM | 59.3 | 41.3 | 0.25 |
|  |  |  | wsnp_Ex_c2171_4073721 | 708 | 3.47 | Full | MLM | 59.3 | 41.3 | 0.25 |
|  | ***QYld.nmbu-7B*** | **701-703** | **BS00083578_51** | 701 | 9.85 | Full/Adapted | FarmCPU, MLM | 48.3 | 12.8 | 0.10 |
|  |  |  | BobWhite_c12048_145 | 701 | 3.53 | Full/Adapted | MLM | 26.8 | 10.7 | 0.37 |
|  |  |  | BS00022522_51 | 701 | 5.24 | Full/Adapted | MLM | 46.1 | 11.8 | 0.10 |
|  |  |  | Excalibur_c50612_146 | 701 | 3.08 | Adapted | MLM | 31.5 | 21.6 | 0.11 |
| PH | ***QHt.nmbu-2A*** | **524-543** | AX-110920715 | 524 | 3.47 | Full | MLM | 3.73 | 9.56 | 0.09 |
|  |  |  | AX-95095516 | 528 | 3.02 | Adapted | FarmCPU, MLM | 4.01 | 12.6 | 0.08 |
|  |  |  | AX-158561839 | 528 | 2.96 | Full/Adapted | MLM | 4.09 | 10.7 | 0.08 |
|  |  |  | **AX-110399256** | 543 | 4.78 | Full | FarmCPU, MLM | 1.81 | 11.6 | 0.08 |
|  |  |  | AX-158540522 | 543 | 2.99 | Adapted | MLM | 4.00 | 12.6 | 0.08 |
|  | ***QHt.nmbu-4A*** | **570-603** | **CAP11_c3631_75** | 570 | 7.52 | Adapted | FarmCPU, MLM | 1.98 | 6.97 | 0.22 |
|  |  |  | RAC875_c19303_228 | 578 | 5.73 | Full/Adapted | MLM | 2.02 | 7.70 | 0.33 |
|  |  |  | AX-95086844 | 603 | 3.14 | Full | FarmCPU | 0.89 | 0.38 | 0.33 |
|  | ***QHt.nmbu-4B*** | **13-59** | AX-158583339 | 13 | 3.43 | Full | MLM | 4.09 | 10.7 | 0.24 |
|  |  |  | BS00037094_51 | 13 | 3.25 | Full | MLM | 1.83 | 5.15 | 0.24 |
|  |  |  | Tdurum_contig93710_409 | 13 | 3.56 | Full | MLM | 1.79 | 4.70 | 0.23 |
|  |  |  | AX-110588223 | 23 | 3.96 | Full/Adapted | MLM | 1.84 | 7.08 | 0.44 |
|  |  |  | wsnp_Ex_c7362_12622736 | 23 | 3.40 | Adapted | MLM | 1.86 | 8.01 | 0.34 |
|  |  |  | **TG0010a; TG0010b** | 31 | 5.50 | Full/Adapted | FarmCPU | 2.87 | 14.6 | 0.28 |
|  |  |  | AX-94685504 | 31 | 5.65 | Full/Adapted | MLM | 2.66 | 15.1 | 0.49 |
|  |  |  | AX-89380014 | 32 | 3.84 | Full/Adapted | MLM | 2.29 | 10.7 | 0.39 |
|  |  |  | AX-111081978 | 36 | 5.82 | Full/Adapted | MLM | 2.61 | 12.2 | 0.30 |
|  |  |  | AX-158564543 | 37 | 6.10 | Full/Adapted | MLM | 2.60 | 12.0 | 0.29 |
|  |  |  | AX-158564633 | 37 | 4.34 | Full/Adapted | MLM | 1.82 | 6.86 | 0.42 |
|  |  |  | AX-158537142 | 38 | 6.42 | Full/Adapted | MLM | 2.63 | 12.2 | 0.29 |
|  |  |  | AX-158564542 | 38 | 5.55 | Full/Adapted | MLM | 2.02 | 7.68 | 0.33 |
|  |  |  | Tdurum_contig33737_157 | 38 | 6.04 | Full/Adapted | MLM | 2.53 | 11.2 | 0.29 |
|  |  |  | IAAV971 | 41 | 7.00 | Full/Adapted | MLM | 2.76 | 12.5 | 0.26 |
|  |  |  | AX-158618805 | 52 | 6.12 | Full/Adapted | MLM | 2.23 | 8.02 | 0.25 |
|  |  |  | Excalibur_c56787_95 | 59 | 6.61 | Full/Adapted | MLM | 2.71 | 11.4 | 0.24 |
|  | ***QHt.nmbu-4D*** | **19-26** | TG0011b | 19 | 25.89 | Full (MLM) Adapted | FarmCPU, MLM | 3.45 | 22.0 | 0.25 |
|  |  |  | TG0011a | 19 | 10.53 | Full/Adapted | MLM | 2.53 | 8.97 | 0.22 |
|  |  |  | **BobWhite_s64797_152** | 26 | 26.06 | Full, Adapted (MLM) | FarmCPU, MLM | 3.18 | 11.7 | 0.29 |
|  | ***QHt.nmbu-6B*** | **202** | **Ra_c10469_616** | 202 | 6.68 | Full | FarmCPU | 2.00 | 5.21 | 0.19 |

**Table S7** Validation of associated markers using sets of independent lines in four seasons in two locations. V – Vollebekk, S – Staur, for instance 2019_S indicates field season 2019 in Staur. Validation results coded as follows: “-“ – phenotype data not available, Mono – marker monomorphic (validation not possible), Rare - minor allele frequency < 0.05, validation not reliable, Rare* - marker with minor allele frequency < 0.05, but visible signs of possible association, Ns – marker polymorphic in the population (minor allele frequency > 0.05), but no significant effect (p <= 0.05), number and unit - marker polymorphic in the population (minor allele frequency > 0.05) and visible significant effect (p <= 0.05)

| QTL | Span | Marker | Pos | Alleles  Pos/Neg | Dominant allele | Validation results | | | | | |
| --- | --- | --- | --- | --- | --- | --- | --- | --- | --- | --- | --- |
|  |  |  |  |  |  | **2019_S** | **2019_V** | **2020_S** | **2020_V** | **2021_V** | **2022_V** |
| *QHd.nmbu-1B* | **1-2** | **BS00022180_51** | 1 | T/C | C | Rare | Rare | - | Ns | Rare* | Rare |
|  |  | BS00071161_51 | 2 | - | T | Rare | Rare | - | Rare | Rare | Rare |
| *QHd.nmbu-7B* | **606** | **BobWhite_c3541_152** | 606 | G/A | G | Rare | Rare | - | Rare* | Rare | Rare* |
| *QMat.nmbu-6B* | **132-136** | wsnp_Ex_rep_c102159_87386822 | 131 | G/A | G | - | 1.29dss | - | 2.23dss | 2.19dss | 0.94dss |
|  |  | **Kukri_rep_c71420_511** | 132 | A/G | A | - | 1.19dss | - | 2.33dss | 2.32dss | 0.89dss |
|  |  | Tdurum_contig14559_741 | 132 | G/A | G | - | 0.72dss | - | 1.27dss | 1.92dss | 0.95dss |
|  |  | Tdurum_contig14559_78 | 132 | G/A | A | - | 0.74dss | - | 1.27dss | 1.89dss | 0.95dss |
|  |  | wsnp_Ex_c12577_20022294 | 132 | - | C | - | Ns | - | Ns | Ns | Ns |
|  |  | GENE-3171_203 | 135 | C/T | C | - | Ns | - | 1.83dss | 2.38dss | 1.72dss |
|  |  | GENE-4074_537 | 135 | G/A | G | - | Ns | - | 1.52dss | Rare | Rare |
|  |  | AX-158529874 | 136 | G/A | G | - | Ns | - | 1.11dss | 1.85dss | 0.78dss |
|  |  | AX-158552731 | 136 | G/A | G | - | Ns | - | 1.70dss | 2.38dss | 1.72dss |
|  |  | Tdurum_contig11700_1247 | 136 | T/C | T | - | Ns | - | 1.10dss | 1.83dss | 0.92dss |
| *QMat.nmbu-6D* | **6** | **BS00022523_51** | 6 | A/G | A | - | Ns | - | - | - | 1.61dss |
| *QYld.nmbu-3A* | **267** | **BS00110129_51** | 267 | A/G | A | Ns | Ns | 35.41g | - | - | 27.71g |
| *QYld.nmbu-5A* | **683-708** | AX-158558760 | 685 | A/G | G | Ns | Ns | Ns | - | - | 18.50g |
|  |  | AX-94387378 | 686 | A/G | G | Ns | Ns | Ns | - | - | 18.52g |
|  |  | AX-95097524 | 694 | G/A | G | 47.63g | Ns | Ns | - | - | Rare |
|  |  | **BobWhite_c8266_227** | 699 | - | - | Mono | Mono | Mono | - | - | Mono |
|  |  | AX-158550750 | 708 | - | A | Ns | Rare | Rare | - | - | Ns |
|  |  | wsnp_Ex_c2171_4072774 | 708 | - | C | Rare | Rare | Rare | - | - | Ns |
|  |  | wsnp_Ex_c2171_4072995 | 708 | - | A | Ns | Ns | Rare | - | - | Ns |
|  |  | wsnp_Ex_c2171_4073721 | 708 | - | T | Rare | Rare | Rare | - | - | Ns |
| *QYld.nmbu-7B* | **701-703** | **BS00083578_51** | 701 | T/C | T | Rare* | Rare | Rare* | - | - | Mono |
|  |  | BobWhite_c12048_145 | 701 | A/C | A | Ns | Ns | 21.56g | - | - | 23.62g |
|  |  | BS00022522_51 | 701 | C/T | C | Rare | Rare | Rare* | - | - | Rare |
|  |  | Excalibur_c50612_146 | 701 | C/T | C | Rare | Rare | 33.81g | - | - | Rare* |
| *QHt.nmbu-2A* | **524-543** | AX-110920715 | 524 | G/A | A | Mono | Rare | Rare | Rare | Rare | Rare* |
|  |  | AX-95095516 | 528 | A/C | A | Mono | Rare | Rare | Rare | Rare | Rare* |
|  |  | AX-158561839 | 528 | - | C | Mono | Rare | Rare | Rare | - | Rare |
|  |  | **AX-110399256** | 543 | - | C | Mono | Rare | Rare | Rare | Rare | Rare |
|  |  | AX-158540522 | 543 | - | C | Mono | Rare | Rare | Rare | Rare | Rare |
| *QHt.nmbu-4A* | **570-603** | **CAP11_c3631_75** | 570 | T/C | T | 7.23cm | 6.41cm | 3.87cm | 4.81cm | 6.53cm | 7.07cm |
|  |  | RAC875_c19303_228 | 578 | A/G | A | 7.24cm | 6.98cm | 3.89cm | 5.53cm | 6.77cm | 8.02cm |
|  |  | AX-95086844 | 603 | C/T | T | Ns | 1.89cm | Ns | Ns | Ns | Ns |
| *QHt.nmbu-4B* | **13-59** | AX-158583339 | 13 | G/A | G | 7.49cm | 4.65cm | 2.94cm | 4.14cm | 5.74cm | 5.48cm |
|  |  | BS00037094_51 | 13 | A/G | A | 7.04cm | 4.42cm | 2.95cm | 3.75cm | 5.57cm | 5.55cm |
|  |  | Tdurum_contig93710_409 | 13 | A/G | A | 7.49cm | 4.38cm | 3.01cm | 3.84cm | 5.66cm | 5.93cm |
|  |  | AX-110588223 | 23 | G/T | G | Ns | 1.98cm | 2.30cm | 2.15cm | 1.96cm | 3.89cm |
|  |  | wsnp_Ex_c7362_12622736 | 23 | T/G | T | Ns | 2.07cm | 2.21cm | 1.97cm | 1.82cm | 3.26cm |
|  |  | **TG0010a; TG0010b** | 31 | C/T | C | 9.36cm | 7.89cm | 4.21cm | 5.73cm | 6.99cm | 8.05cm |
|  |  | AX-94685504 | 31 | G/A | Unclear | Ns | 5.70cm | 4.23cm | 4.68cm | 5.60cm | 6.80cm |
|  |  | AX-89380014 | 32 | G/A | Unclear | 6.25cm | 6.04cm | 4.01cm | 4.01cm | 5.06cm | 5.69cm |
|  |  | AX-111081978 | 36 | C/T | C | 6.43cm | 6.89cm | 4.08cm | 5.44cm | 6.80cm | 7.84cm |
|  |  | AX-158564543 | 37 | A/G | A | 7.24cm | 7.04cm | 4.02cm | 5.48cm | 6.72cm | 8.02cm |
|  |  | AX-158564633 | 37 | A/G | G | 6.65cm | 4.33cm | 2.22cm | 3.63cm | 1.50cm | 2.43cm |
|  |  | AX-158537142 | 38 | C/T | C | 7.24cm | 7.01cm | 3.88cm | 5.40cm | 6.73cm | 7.99cm |
|  |  | AX-158564542 | 38 | A/C | A | 7.24cm | 7.01cm | 3.86cm | 5.51cm | 6.67cm | 8.02cm |
|  |  | Tdurum_contig33737_157 | 38 | A/G | A | 7.24cm | 6.98cm | 3.95cm | 5.54cm | 6.74cm | 8.02cm |
|  |  | IAAV971 | 41 | C/T | C | 7.24cm | 6.97cm | 4.07cm | 5.38cm | 6.60cm | 7.96cm |
|  |  | AX-158618805 | 52 | C/T | C | 7.24cm | 6.56cm | 3.99cm | 4.88cm | 6.50cm | 6.69cm |
|  |  | Excalibur_c56787_95 | 59 | G/T | G | 7.24cm | 6.74cm | 4.11cm | 5.18cm | 6.70cm | 6.72cm |
| *QHt.nmbu-4D* | **19-26** | TG0011b | 19 | G/T | G | Mono | 5.59cm | Ns | 4.76cm | 6.14cm | 10.44cm |
|  |  | TG0011a | 19 | G/T | G | Mono | 4.76cm | 3.29cm | 5.66cm | 7.46cm | 10.40cm |
|  |  | **BobWhite_s64797_152** | 26 | A/C | A | 7.24cm | 7.01cm | 3.85cm | 5.50cm | 6.72cm | 8.03cm |
| *QHt.nmbu-6B* | **202** | **Ra_c10469_616** | 202 | G/A | A | Rare | Rare | 2.39cm | 2.24cm | Ns | 4.32cm |

**Figure S24** Locus associated with adaptation to the Norwegian growing conditions: *QYld.nmbu-5A*. Allele frequencies in the full panel and adapted and exotic parts (a), haplotype analysis of the loci (b) on days to heading (DH)). Comparison among the alleles was performed using Tukey’s HSD test. Alleles with the same letter are not significantly different (α = 0.05). Alleles with low frequencies (in less than ten lines) were gathered into the “Other” bin
